# Small eye movements cannot be reliably measured by video-based P-CR eye-trackers

**DOI:** 10.3758/s13428-020-01363-x

**Published:** 2020-03-23

**Authors:** Kenneth Holmqvist, Pieter Blignaut

**Affiliations:** 1 Institute of Psychology, Nicolaus Copernicus University in Torun, Toruń, Poland; 2 Department of Psychology, Regensburg University, Regensburg, Germany; 3 Department of Computer Science and Informatics, University of the Free State, Bloemfontein, South Africa; 4 Faculty of Arts, Masaryk University, Brno, Czech Republic

**Keywords:** Eye-tracker, Data quality, Resolution, Precision, Microsaccade, Corneal reflection, Saccade amplitude

## Abstract

For evaluating whether an eye-tracker is suitable for measuring microsaccades, Poletti & Rucci (2016) propose that a measure called ‘resolution’ could be better than the more established root-mean-square of the sample-to-sample distances (RMS-S2S). Many open questions exist around the resolution measure, however. Resolution needs to be calculated using data from an artificial eye that can be turned in very small steps. Furthermore, resolution has an unclear and uninvestigated relationship to the RMS-S2S and STD (standard deviation) measures of precision (Holmqvist & Andersson, 2017, p. 159-190), and there is another metric by the same name (Clarke, Ditterich, Drüen, Schönfeld, and Steineke 2002), which instead quantifies the errors of amplitude measurements. In this paper, we present a mechanism, the Stepperbox, for rotating artificial eyes in arbitrary angles from 1^′^ (arcmin) and upward. We then use the Stepperbox to find the minimum reliably detectable rotations in 11 video-based eye-trackers (VOGs) and the Dual Purkinje Imaging (DPI) tracker. We find that resolution correlates significantly with RMS-S2S and, to a lesser extent, with STD. In addition, we find that although most eye-trackers can *detect* some small rotations of an artificial eye, the rotations of amplitudes up to 2^∘^ are frequently erroneously *measured* by video-based eye-trackers. We show evidence that the corneal reflection (CR) feature of these eye-trackers is a major cause of erroneous measurements of small rotations of artificial eyes. Our data strengthen the existing body of evidence that video-based eye-trackers produce errors that may require that we reconsider some results from research on reading, microsaccades, and vergence, where the amplitude of small eye movements have been measured with past or current video-based eye-trackers. In contrast, the DPI reports correct rotation amplitudes down to 1^′^.

## Introduction

**Fig. 1:**
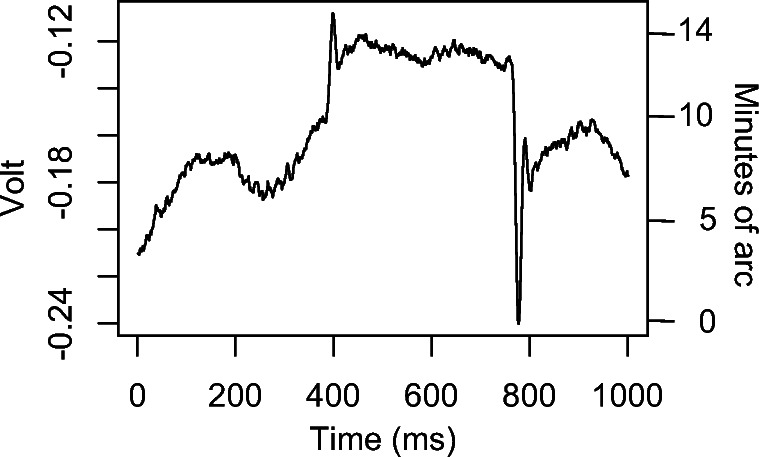
Part of a fixation with two microsaccades, 3^'^ and 7^′^ in amplitude, embedded in oculomotor drift, recorded with a DPI. Which eye-trackers can reliably detect and measure eye movements of this amplitude?

The *precision* of an eye-tracker is one of the most important aspects of its quality (Holmqvist & Andersson, 2017, p. 170-190). Loosely speaking, the precision of an eye-tracker is a measurement of the amount of noise in the data. More formally, precision could be defined as the ability of the eye-tracker to reliably reproduce a gaze position measurement, from one sample to the next, assuming a stable true gaze position. Two measures of precision are commonly used. First, RMS-S2S is a measure of the velocity of the noise (assuming sampling frequency is known), calculated as the root mean square of the sample-to-sample movement. Second, STD measures the spatial extent of the noise; here we will calculate STD as the square root of the pooled variance of X and Y. Precision values give an indication of the signal to noise ratio, and predict how well an algorithm could perform the detection of events such as fixations and saccades in that data. Events with a small extent (such as the microsaccades in Fig. 1), referred to as amplitude in the case of saccades, are more likely to be hidden and go undetected in data with poor precision (Holmqvist, Nyström, & Mulvey 2012). Even data analysis with areas of interest will be affected by poor precision (Orquin & Holmqvist, 2017). Next to accuracy and sampling frequency, precision is the most likely property to appear in the specification sheets from eye-tracker manufacturers.

The *resolution* of an eye-tracker is often described as the smallest reliably detectable eye movement for that eye-tracker (Holmqvist & Andersson, 2017, p. 189-190); (Poletti & Rucci, 2016). Unless otherwise stated, this is the definition of resolution to which we refer. Resolution in the sense of the smallest reliably detectable movement appears to be closely related to precision, to the extent that resolution has been used interchangeably with RMS-S2S, as exemplified by Table 1. Indeed, a link between the two measures would seem natural, assuming that the resolution of an eye-tracker is determined by the noise: Increasing noise, as measured by RMS-S2S, would naturally drown out ever larger movements and conceal them from detection (Holmqvist et al., 2012), and thus the level of noise would tell us the smallest amplitude movement that would not be drowned out. For this reason, although the precision and resolution describe very different properties of the signal, the measures quantifying the two properties could be expected to correlate.

**Table 1:** Reproduced from part of the EyeLink Manual v 1.52, page 9, which uses the term resolution for the precision measure RMS-S2S, here is referred to as simply RMS

Spatial Resolution	<0.01^∘^ RMS	1000 Hz	<0. 1^∘^ RMS

The existence of a strong relationship between resolution and precision has been questioned by Poletti and Rucci (2016), who argue that RMS-S2S does not adequately represent the resolution of eye-trackers: RMS-S2S “is only marginally informative. It does not represent the system’s resolution, but the ideal limit in resolution which could be achieved in theory” and furthermore point out that a “system with a huge quantization step would give very little noise, but would obviously also be poorly sensitive.” (Poletti & Rucci, 2016, p. 87). This critique of RMS-S2S could be seen as our starting point to the current study. Quantization refers to reducing the set of possible values in a signal, for instance by rounding or truncation, which is part of all analog-to-digital conversions, for instance in the eye camera of eye-trackers.

In contrast, EyeLink representatives have presented the opinion that “When it comes to microsaccade research, detection is exclusively influenced by precision,”^1^ which in our interpretation contradicts the argument by Poletti and Rucci (2016) that resolution is poorly represented by the RMS-S2S of the eye-tracker when it comes to microsaccade detection. If the ability to detect microsaccades is indeed better predicted by other metrics of the eye-tracker, such as Poletti and Rucci suggest, then it might be interesting for SR Research and other companies to give weight to those metrics in their future product development and benchmarking.

On a more conceptual level, Clarke et al. (2002) present an alternative definition of measurement resolution. They rotate an artificial eye over a distance of 4^∘^ in increments of 0. 1^∘^. For each step they calculate the measured movement amplitude. The difference between the known eye movement (0. 1^∘^) and the measured eye movement constitutes an amplitude error in the measurement. The standard deviation of these errors is defined to be the measurement resolution. This is another definition of resolution, and it is unclear how it relates to the definition above by Holmqvist & Andersson, and Poletti and Rucci.

For oscillatory movements (tremor), resolution of the eye-tracker can be based on a power spectral density calculation (a PSD). For an eye-tracker capable of measuring tremor, we typically find a peak around 90 Hz in human data that is absent in data recorded with artificial eyes (Ko, Snodderly, & Poletti 2016). This is a definition of resolution that we will not address.

*Linearity* measures the ability of an eye-tracker to reproduce the same amplitude and direction reliably throughout a measurement space, such as a monitor display ( Holmqvist & Andersson (2017, p. 190); Clarke et al. (2002); McConkie (1981)). Measurement errors would lead to non-linear data, which in turn implies offsets in gaze data, known as inaccuracy. However, non-linear data also make detection of events more unreliable, for instance where the erroneously recorded movement amplitudes and velocities fall below the algorithm threshold, although the actual eye movement does not.

Very few studies have reported resolution of eye-trackers. Notable exceptions are Crane and Steele (1985) and Clarke et al. (2002), whose methods involve rotating an artificial eye in steps of a known amplitude. This allowed these authors to state that movements of at least the amplitude used (1^′^ and 0. 1^∘^, respectively) can be detected and measured by the eye-tracker. The data plots in both these papers clearly suggest that even smaller movements could very well be detected. Since the definition above of resolution is the *minimum amplitude* at which movements are reliably detected, it follows that the values reported by both Crane and Steele (1985) and Clarke et al. (2002) are not the minimum resolution values but values larger than the minimum resolution amplitude.

Both definitions of minimum resolution require that an artificial eye can be rotated at arbitrary angles from, for instance, 1^′^ and above. With access to such data, we could determine from x-t plots showing many different step sizes, at which minimum amplitude those rotations can be seen against the noise. If this could be done for a large number of eye-trackers, we could look for the correlation between resolution and precision, and possibly show how to calculate the signal resolution from the RMS-S2S or STD values of those eye-trackers.

In this paper, we set out to investigate this relationship between precision and resolution. We present a custom-built mechanism for measuring resolution, defined either as the minimum detectable amplitude in video eye-trackers, or as the standard deviation of the amplitude errors. We provide signal resolution measurements according to both definitions for the eye-trackers listed in Table 1.

We differ from previous research in three important aspects. First, Clarke et al. (2002), and the DPI measurements by Ko et al. (2016), Poletti and Rucci (2016), and Crane and Steele (1985) all aim to show that their respective eye-tracker has a resolution better than the step size (0. 1^∘^ or 1^′^) of the movement of the artificial eye, but they do not report the actual resolution, which is lower than the step size they use. This requires a mechanism that can turn artificial eyes in steps of close to arbitrary amplitudes.

Second, following Clarke et al. (2002), but differing from Ko et al. (2016), Poletti and Rucci (2016), and Crane and Steele (1985), we will rotate the artificial eyes in sequences of steps across a larger area, not just in a square wave, so that we can calculate values for both definitions of resolution.

Third, all previous methods for determining measurement resolution in eye-trackers seem to have been developed by manufacturers (Clarke et al. 2002; Crane & Steele 1985) and employed only on their own systems. As far as we are aware, no one has ever before published comparative resolution values from many different eye-trackers.

## Method for measuring eye-tracker resolution

### Design

Our method for determining the minimal resolution of an eye-tracker is based on the decision of four experts visually assessing plots of our data, blinded to each other’s judgements. Our choice of using visual assessment for determining the minimal resolution was inspired by plots of square-wave signals recorded by the DPI eye-tracker (1^′^), for instance in Fig. 4 of Ko et al. (2016), Fig. 2 of Poletti and Rucci (2016), and Fig. 7 of Crane and Steele (1985), used as indicators of resolution.

However, we also calculate the recorded step sizes in the reported eye-movement data in order to be able to report the standard deviation of the errors in movement amplitudes, following Clarke et al. (2002). This allows us to investigate the relation between precision measures and measures for the two contending definitions of resolution.

### Apparatus

**Fig. 2:**
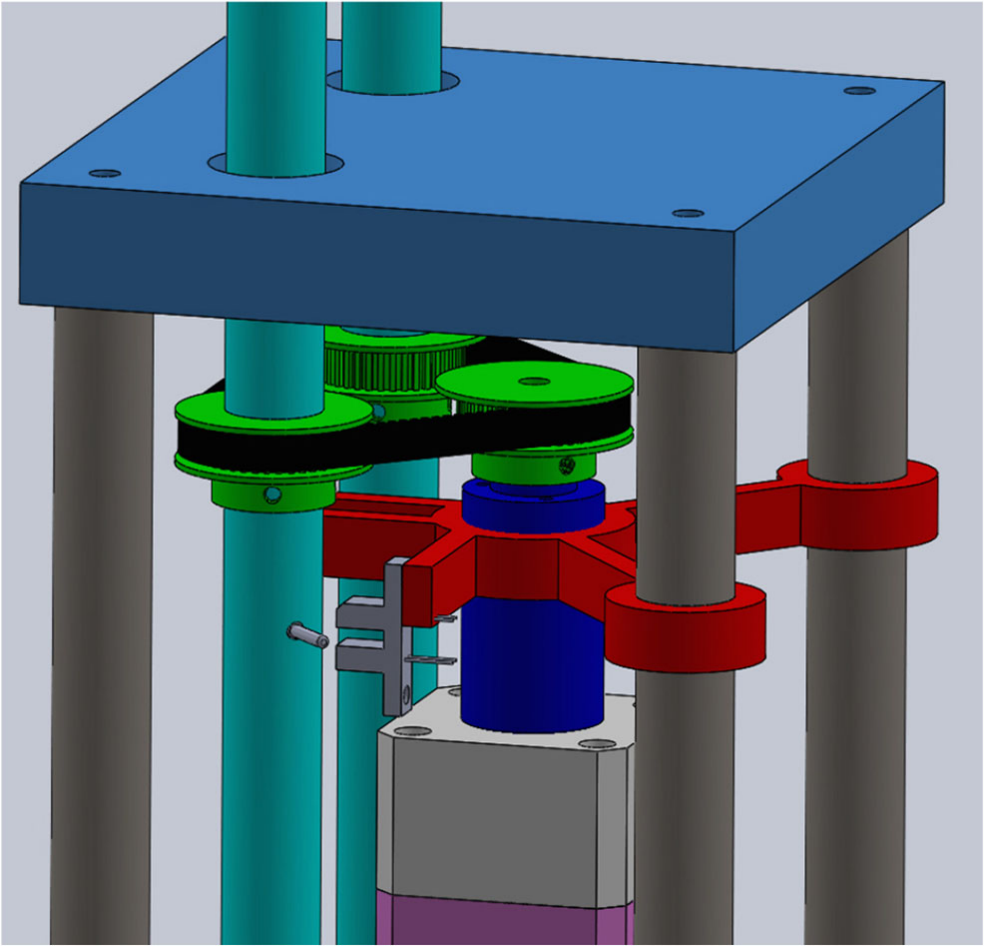
View of part of the interior mechanism of the Stepperbox. A toothed rubber belt in a triangle configuration between the motor and the two shafts makes both shafts (and hence eyes) move simultaneously.

**Fig. 3:**
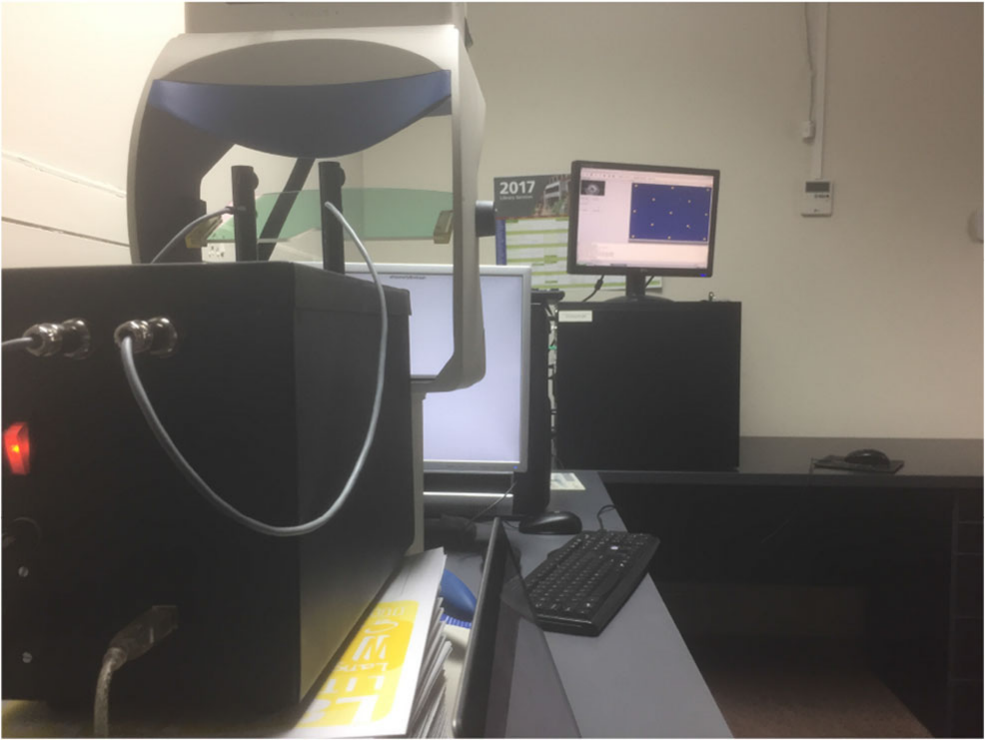
The mechanism for turning artificial eyes in a recording with an SMI HiSpeed 1250 eye tracker.

In order to measure resolution and errors in registered movement amplitudes, we built an electro-mechanical instrument that turns a pair of artificial eyes (Figs. 2 and 3) in discrete steps down to 3 ′ ′ (arcsec). It is built with a stepper motor and a gearbox as central parts, which drive two shafts that extend up through the roof of the box, on which two artificial eyes can be attached. The radius of the shafts was chosen so that the rotations of the artificial eyes have the same rotation radius as human eyes. A toothed rubber belt in a triangle between the motor and the two shafts connects all three, so that both shafts rotate through the same angle at exactly the same time.

The Stepperbox rotations do not have the velocity profile of human saccades, in contrast to artificial saccade generators such as those presented, for instance, by Reingold (2014). The movements in our Stepperbox have the same velocity throughout the whole movement, for all step sizes. The speed resembles slow smooth pursuit (0.845^∘^/s). We chose to prioritise the ability to make very small movements with a high systematicity over saccade-like velocity in steps, as the latter is irrelevant to measuring step sizes. This steady, slow movement allows us to investigate the detectability and errors of step sizes equally well as if the Stepperbox were to make jerky, saccade-like movements. However, the construction of a slow system is much easier than it would be to build a mechanical system that mimics the velocity profile of human saccades of any given amplitude.

Artificial eyes (described below) were mounted onto the shafts using a combination of velcro, poster putty and electric isolation tape, that was renewed for each new recording. We took care to make sure that eyes were rigidly attached and found no evidence in data to the contrary.

**Fig. 4:**
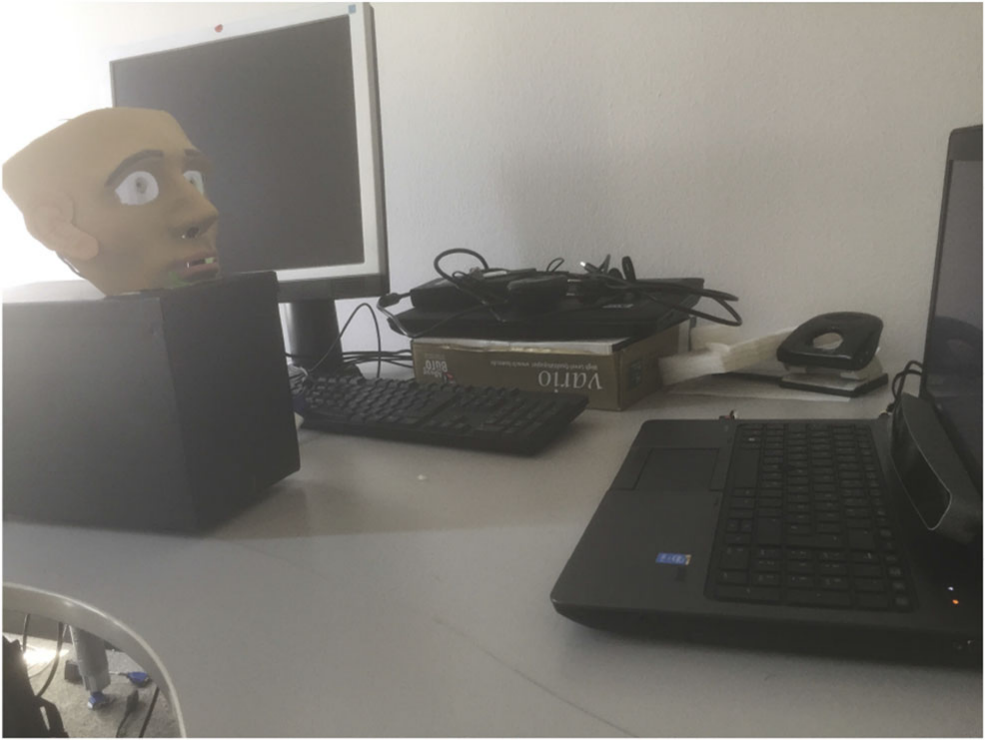
The SMI RED250mobile and the Tobii Spectrum only tracked the artificial eyes if they were embedded in a face mask.

The Tobii Spectrum and the SMI RED250mobile did not track artificial eyes unless there was a face mask around the eyes. After some trial and error, we found a face mask that worked somewhat with both trackers. Figure 4 shows the mask in use with the SMI RED250mobile. Holes for the eyes were made large enough so that the artificial eyes could move freely without interference from the mask. The border between mask and artificial eyes could have influenced noise levels in part of the data for the Spectrum, but we deemed that the parts of the data used for resolution measurements were sufficiently precise.

Wherever filters could be set on or off, that is, on many of the SMIs and on the EyeLinks, we left them at the default (on). Some of these eye-trackers only provide calibrated gaze samples (*x*, *y*, *t*), while others, specifically the SMIs and the EyeLinks, also provide pre-calibrated coordinates of the centres of the pupil and the corneal reflection(s) in the eye camera. The DPI outputs a signal for its corneal reflection, known as ‘the 1st’ in DPI terminology, and the unique 4th Purkinje, but no pupil data. In this paper we will refer to the signal representing the centre of the corneal reflection in the camera as the CR signal, while the Pupil (or P) signal represents the centre of the pupil in the eye camera.

The importance of using the Pupil and CR signals from the eye-tracker both for validation and for studying data quality stems from the fact that in the P-CR eye-trackers of Table 2, the movement in the Gaze signal is strictly determined by the movements of the P and CR signals. Errors in either the Pupil or the CR signals will by necessity give errors in the Gaze signal. In all the following plots where CR and Pupil are shown next to Gaze, we scale the CR and Pupil signals so they are both in degrees, following Hooge, Hessels, and Nyström (2016).

**Table 2:** The eye-trackers. The 1st Purkinje and the CR (corneal reflection) refer to the same infra-red reflection in the cornea. The 4th Purkinje is a reflection in the back of the human lens. The P-CR method calculates uncalibrated gaze as the difference between the centres of the pupil image and the CR image in each video frame of the eye. We also recorded the EyeLink II in pupil-only mode, a predominant setting when measuring micro-saccades, and will report those data later. Polynomial calibration uses the solution of a higher-order polynomial equation system to map P and CR data to gaze, while 3D modelling of the eye estimates parameters of the shape of the eyeball.

Eye tracker	Interface	Method	Calibration	Sampling frequency (Hz)
DPI Gen5.5	Head fixed	Analog, 1st and 4th Purkinje	Custom	1000
SMI HiSpeed 240	Head fixed	P-CR video, filtered	Polynomial	240
SMI HiSpeed 1250	Head fixed	P-CR video, filtered	Polynomial	500
SMI RED250	Remote	P-CR video, filtered	Polynomial	250
SMI RED250mobile	Remote	P-CR video, filtered	Polynomial	250
SMI ETG 2	Glasses	P-CR video, filtered	3D model	60
SR Research EyeLink II	Head fixed	P-CR video, filtered (standard)	Polynomial	250
SR Research EyeLink 1000+	Remote	P-CR video, filtered (standard)	Polynomial	500
Tobii X2-60	Remote	P-CR video, unfiltered	Polynomial	60
Tobii T120	Remote	P-CR video, unfiltered	Polynomial	120
Tobii TX 300	Remote	P-CR video, unfiltered	3D model	300
Tobii Spectrum	Remote	P-CR video, unfiltered	3D model	600

### Validation of the measurement method

For validating the rotation amplitudes of the Stepperbox, we first used a 25 meter-long corridor. At one end, we placed the Stepperbox with a laser pointer attached to each shaft and then marked the position of the laser dot on the wall at the other side of the corridor. We also marked the positions after a 1^′^ movement to the left. A total of 13 such measurements were made, and 11 identical movements to the right. In this and all following analyses, slack-compensation movements were included, and we additionally ignored the first two movements in each measurement sequence. For the 1^′^ (0.0167^∘^) movement in the device, we measured an average 0.0151^∘^ (SD 0.00189^∘^) movement of the laser to the left, and 0.0101^∘^ (SD 0.0012^∘^) to the right. To test whether this reduction affects sequences of 1^′^ movements, we also let the mechanism make ten sequences of 30 rightward 1^′^, which gave an average total rotation of 0.474^∘^ (SD 0.002^∘^), which is 5% below the expected 0. 5^∘^ movement.

The same test was done for 10^′^ (0.167^∘^) movements in the device. We measured an average 0.162^∘^ (SD 0.005) movement of the laser dot to the left, and 0.161^∘^ (SD 0.002) to the right, which is a 3% reduction. Sequences of larger amplitudes tended to result in smaller relative errors. For instance, while a sequence of 30 rightward 1^′^ steps gave a 5% error, a later test in an 11 meter room showed that 10 steps of 30^′^ resulted in a rotation less than 2% below the nominal 5^∘^.

**Fig. 5:**
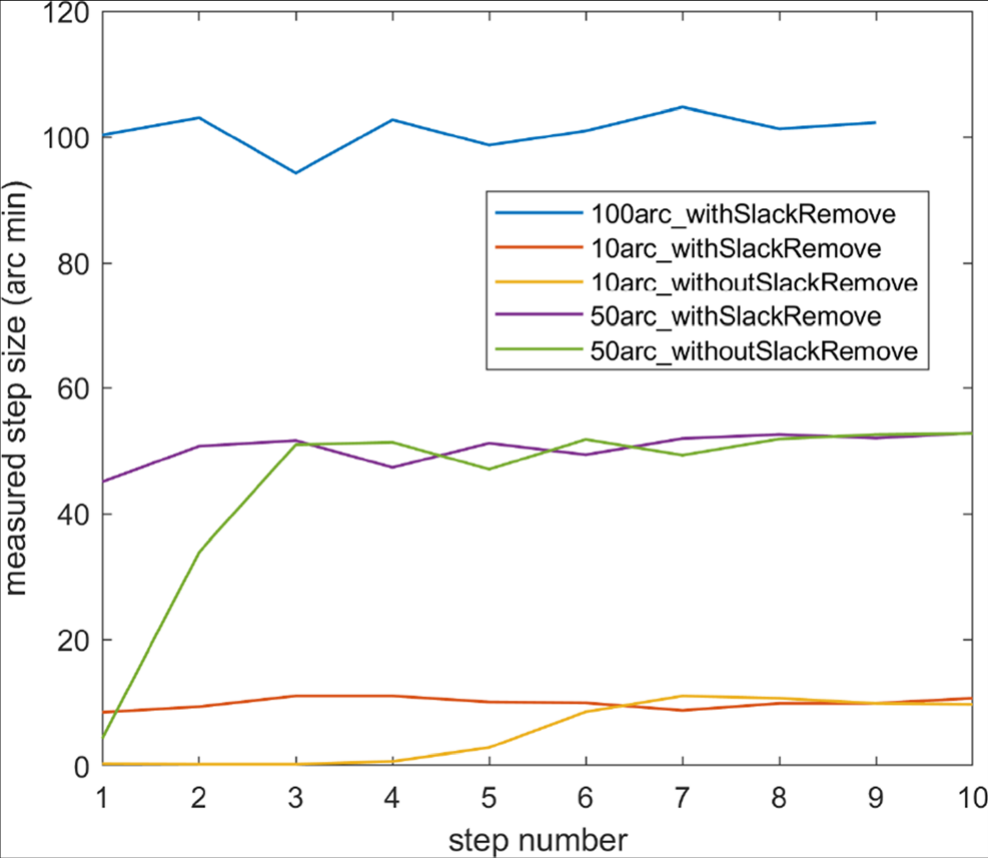
Validation measurements using the laser dots show that the substantial slack in the system can be removed. These also show that the Stepperbox exhibits a certain order error, which is quantified in the text.

A final validation was made on staircase sequences (defined below in Section 2.4) of ten each of 10^′^, 50^′^ and 100^′^ steps. Laser dots with a width of 6 mm were projected onto a measuring tape attached on a wall at a distance of 931.5 cm. The eleven positions of the laser dot for each of the 10 steps were noted with an estimated accuracy of 3 millimeters. Five repetitions were made of each staircase sequence. Results show that there is indeed slack in the system, but that the slack can be consumed by adding four 20^′^ steps before the measurements (Fig. 5).

We then quantified the order errors. For the slack-free measurements, the standard deviation of the 10^′^, 50^′^ and 100^′^ step sizes of the laser dot were 0. 8^′^, 2.47^′^ and 3.01^′^, respectively. Relative errors of the laser were within the ranges -14.5% to +11.5% (mean: 5.7%) for 10^′^ steps, -10.4% to +4.5% (mean: 3.8%) for 50^′^ steps, and -6.6% to +3.2% (mean: 2.2%) for 100^′^ steps. These errors are presented as histograms in Fig. 13(a).

**Fig. 6:**
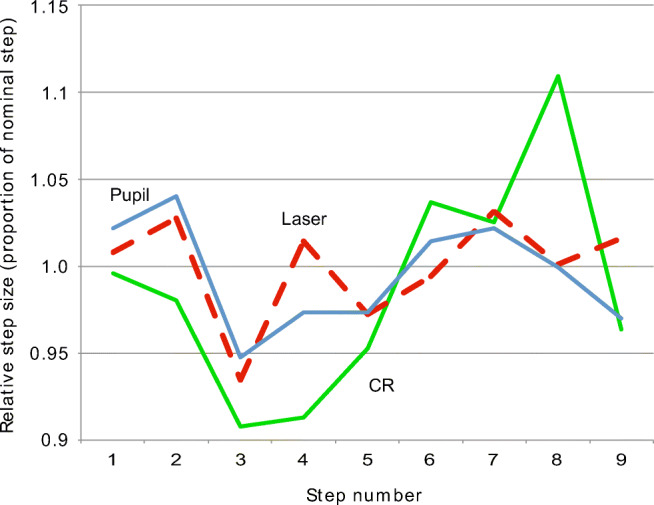
The relative step sizes for 100^′^ steps of the laser in the last validation and Pupil and CR of the EyeLink 1000+, recorded separately. The Pupil signal and the striped laser signal are similar, but the CR, co-recorded with the Pupil signal, has a poorer fit to the laser signal.

These order errors are very systematic through each staircase sequence. The variation in size for the same step, between the five validation repetitions of staircases, exhibited a standard deviation of 0.10^′^, 0.23^′^ and 0.23^′^ for the 10^′^, 50^′^, and 100^′^ steps, respectively. This strong systematicity encouraged us to investigate whether the pupil signal of data recorded with the Stepperbox mechanism on the EyeLink 1000+ would exhibit the same order effect as the laser measurements did, which could be expected as the pupil of the artificial eye is a fixed feature that turns with the shafts of the Stepperbox. Figure 6 indeed exemplifies such a relationship between Pupil and laser (R = 0.69), but a weaker relationship between CR and laser (R = 0.37). We take this as evidence that our laser validation measurement is representative of the measurement with the eye-trackers.

**Fig. 7:**
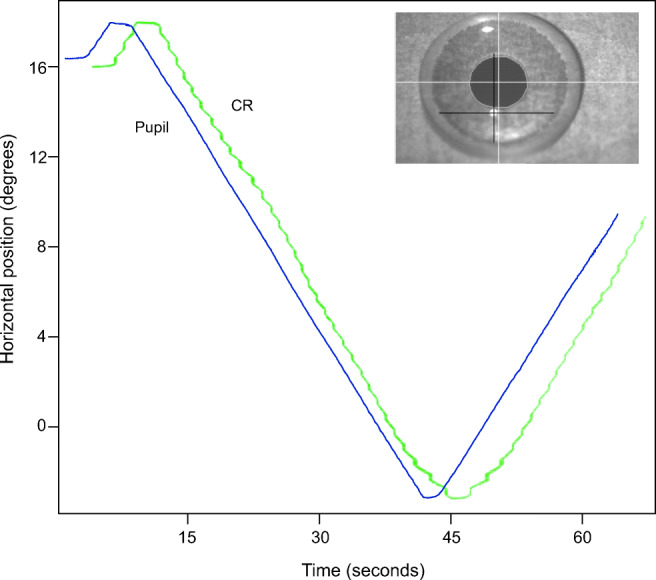
The green CR and blue Pupil signals of the SMI HiSpeed240 during a smooth 19. 5^∘^ continuous sweep for 60 seconds. The CR is shifted in time for visibility. Note how clean the pupil signal is (representing the smooth rotation of the Stepperbox), and how jagged the CR signal is (a repeating artefact). Also note how the artefacts in the CR signal are mirrored in the turning points. Inlaid is the eye video of the HiSpeed240 with the SR Research artificial eye. Here, the pupil diameter spans 77 pixels, while the CR diameter spans approximately 8 pixels, which are common sizes for high-end eye-trackers.

We evaluated smooth, continuous rotations of the Stepperbox mechanism by inspection of plots such as Fig. 7, where a smooth rotation of the Stepperbox indeed results in a smooth scan of the Pupil signal, but not of the CR. Because the pupil feature of an artificial eye is a fixed feature of the eye that moves with the rotation of the Stepperbox, we consider smooth movements to be produced with no noticeable artefacts. The jagged CR signal in Fig. 7 is consistent with the impression in Fig. 6 that the CR is a larger contributor to error than the Pupil signal.

When we report errors in the eye-trackers below, we have considered the errors in the Stepperbox to be so small that for the purposes of this paper, there is no need to compensate for them.

### Scripts for recording resolution data

**Fig. 8:**
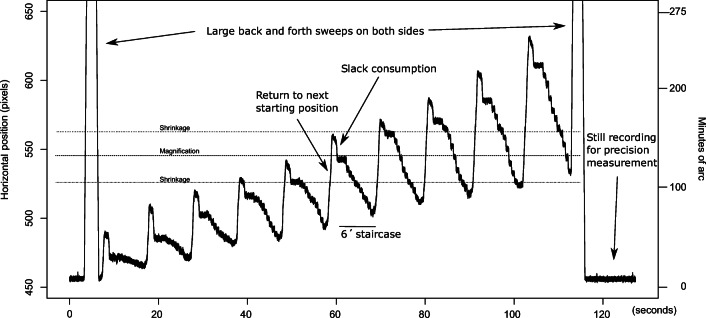
Data plots of this kind were used for visual assessment of the resolution of the eye-trackers. The plot shows EyeLink 1000+ data from the staircase script with 10 steps each of amplitudes 1^′^ to 10^′^. The smallest detectable movements are 3-4 minutes of arc. One amplitude magnification and two shrinkage areas are indicated with dashed lines.

We built three groups of command scripts for controlling the rotations of the artificial eyes. The first group are *staircase rotations*, designed to find the smallest reliably detectable rotation of the eyes. The first staircase script records 10 rotations of 1^′^, then 10 rotations of 2^′^, etc., up to 10 rotations of 10^′^ (a plot of such data can be seen in Fig. 8). We also built one script to make 10 rotations each of amplitudes 2^′^, 4^′^, 6^′^, ... , 20^′^, another to produce 10 movements of amplitudes 10^′^, 20^′^, 30^′^, ... , 100^′^, and finally one script to generate four rotations of amplitudes 60^′^, 90^′^, 120^′^, ..., 330^′^, that is, from 1^∘^ to over 5^∘^. Because 10 rotations of the last script would take gaze coordinates beyond the measurement range (e.g. for 330’, the range would be 330^′^ ∗ 10 = 55^∘^), we generated only four rotations (330^′^ ∗ 4 = 1320^′^ = 22^∘^) at each value of the set. We let the eyes make a 4 times 20^′^ slack consumption movement and then rest still for 4000 ms before a staircase recording started, and for 1000 ms between each rotation in every staircase script. All rotations were done to the left and were symmetrically positioned around the center of the monitor, but the starting position was moved slightly to the right for every new staircase to make sure that the same gaze directions were reached for a different step in each new staircase. The eyes were taken to the next starting position with a rightward continuous sweep after the last rotation in each set of 10 (or four) rotations. A complete set of the 1^′^ to 10^′^ staircases can be seen in Fig. 8.

The second group of movement scripts were *step sequences*, consisting of steps in amplitudes of 8^′^ across the recording area. The purpose of these rotations was to measure whether a rotation amplitude of 8^′^ was measured as 8^′^ everywhere on the monitor, or whether the measured amplitude was somewhere incorrectly reported. Initial tests had shown that 8^′^ steps were locally mismeasured in many eye-trackers, and was deemed a good amplitude for step sequences.

The third movement script recorded continuous *sweeps* in the form of long slow movements at 0.845^∘^/s across large parts of the measurement space (exemplified in Fig. 7).

In addition to the three groups of movement data for the calculation of resolution, we also recorded data from still artificial eyes for 2 minutes on each eye-tracker, for the calculation of the precision measures STD and RMS-S2S.

### Selection of artificial eyes

Tobii AB, SMI GmbH and SR Research have all manufactured their own artificial eyes. We tested all three eyes on the SMI HiSpeed 240 using the following procedure. First, we calibrated the eye-tracker with human eyes, and then positioned the Stepperbox in the eye-tracker so the eye-image was sharp and feature detection of CR and Pupil clear and unambiguous. We recorded data with the 10-100^′^ staircase script using one artificial eye. We then carefully replaced it with the second artificial eye, while making sure that the Stepperbox did not change position, and that the second eye was attached in the same place where the first eye had been. We then recorded with the second and eventually third eye using the same procedure.

We investigated the horizontal center coordinates of the pupil and CR images in the eye camera coordinate system, in order to examine how similar the average coordinate values for all 11 stops were between the three recordings. The average standard deviation of the position values was 0.012 camera pixels for the CR, and 0.004 camera pixels for the pupil, which tells us that the coordinates in each of the three recordings were nearly identical, and that the variation between step amplitudes recorded with different artificial eyes was very small, but that the same also held true for successive runs of the Stepperbox.

**Fig. 9:**
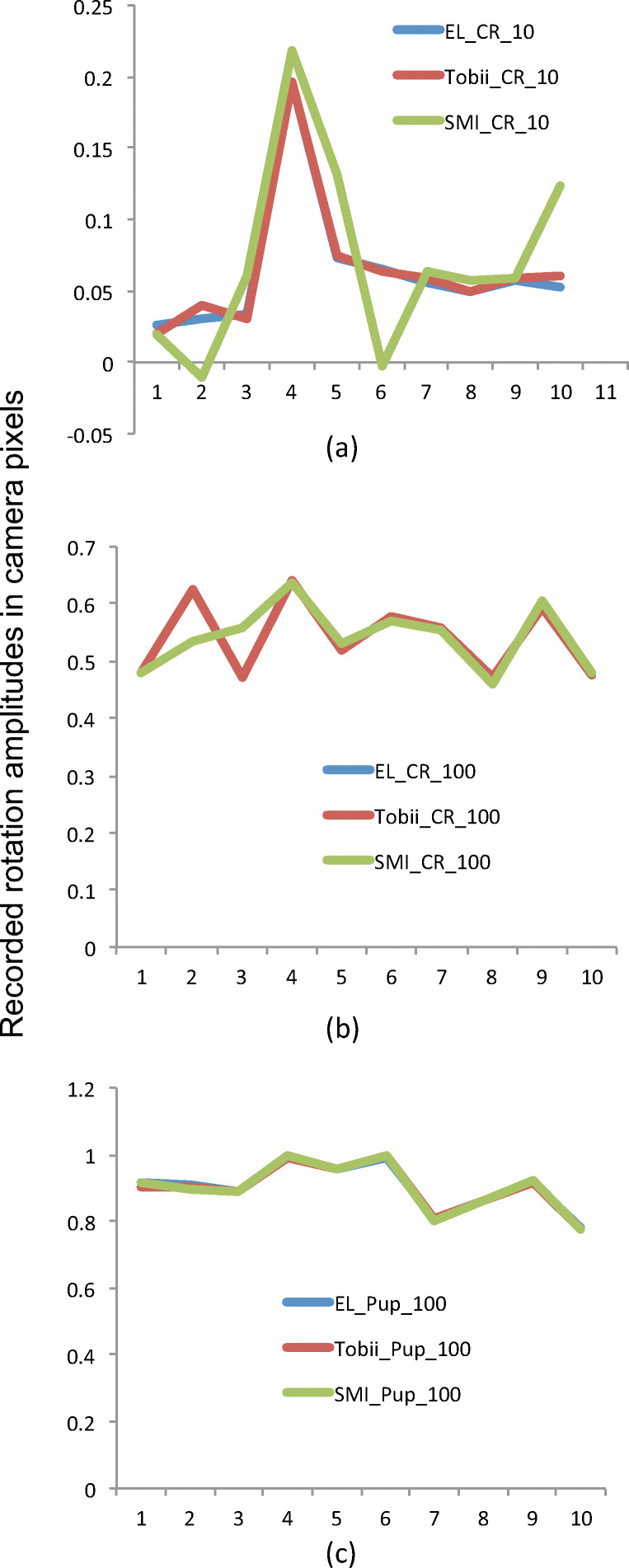
In (a), overlays of the ten recorded 10^′^ step sizes of the CR in camera pixels of the SMI HiSpeed240, for SMI, Tobii and SR eyes. All eyes result in the same large mismeasurement in step 4. In (b), the CR steps for 100^′^ steps. Except for steps 2 and 3, all three eyes result in nearly identical data. In (c), the pupil steps for 100^′^ are indistinguishable between the three artificial eyes.

We then plotted the step sizes of the Pupil and CR signals, which were mostly very similar between eyes, with one or two random exceptions. Figure 9 shows three typical examples of plots. Note that a 10^′^ step corresponds to circa 0.06 pixel movements in the eye camera for this 8-pixel CR, which suggests that camera resolution might be the cause of the CR errors in Figs. 6 and 7.

We concluded that it does not matter for testing amplitude errors which eye we use, and settled for using the SMI eyes, with a fixed 4mm pupil, in all recordings with the video-based eye-trackers. However, for the DPI, we used the model eye that comes with the DPI. This may introduce a small difference, but it is inevitable as the DPI cannot operate with the artificial eyes used by the video-based eye-trackers (because they lack a 4th reflection), and the video-based eye-trackers cannot operate with the DPI model eye (which lacks a pupil).

### Eye trackers

We recorded on the Tobii X2-60, T120, TX 300, and the Tobii Spectrum. We also recorded on the SMI HiSpeed 240, the HiSpeed 1250 at 500 Hz, the RED250, the RED250mobile and the ETG 2 at 60 Hz. Finally, we recorded the SR Research EyeLink 1000+ at 500 Hz, and the DPI with the LabJack U6 1000 Hz AD converter, which claims a 16 bit resolution. This selection was chosen to give us a range from very noisy to quite clean signals. We also recorded on the EyeLink II, which has been extensively used in microsaccade research, as evidenced by Table 1 in Martinez-Conde, Macknik, Troncoso, and Hubel (2009). We failed to record data on the Tobii Glasses in both its generations.

In this selection of eye-trackers, the DPI is the odd one out. It differs from the others in being considered a superior eye-tracker. Specifically, it is not a P-CR video eye-tracker and it does not need to be calibrated. The high maintenance costs and learning thresholds have made researchers abandon it for modern P-CR video eye-trackers, which now entirely dominate the market. However, the DPI does not provide the ground truth in this study. Instead, the Stepperbox laser provides our ground truth.

### Procedure

We first calibrated each eye tracker on a human eye whenever the software demanded it, using the built-in manufacturer software, because this is a prerequisite to recording data in all machines except the DPI. If it had not been for limitations in the software of the other eye-trackers, calibration would not have been necessary at all.

We then calculated, for each recording, ten scaling factors to translate between pixels (whether Gaze, Pupil or CR) and minutes of arc. Each scaling factor is calculated as the average quotient of the recorded data in pixel distance to generated Stepperbox rotation over each of 10 rotations (80-800^′^).

There is a possibility that calibrating on human eyes and then recording on artificial eyes could introduce non-linearities in the polynomials of the calibration matrix, which would produce errors. However, Table 3 shows that the standard deviations of the ten scaling factors per eye-tracker are generally low, which we take as evidence that no large artefacts were introduced by the calibration mapping when switching from human to artificial eye.

**Table 3:** Average scaling factors in the unit pixels per minute of arc (voltages/arcmin for the DPI), over 10 different estimations per eye-tracker. These scaling factors were used to calculate the amplitude errors

Eye tracker	Average	SD
DPI Gen5.5	0.00559	0.000947
SMI HiSpeed 240	0.573	0.0618
SMI HiSpeed 1250	0.511	0.0243
SMI RED250	0.0243	0.00244
SMI RED250mobile	0.746	0.0970
SMI ETG 2	0.180	0.0292
SR Research EyeLink II (P-CR)	0.705	0.0145
SR Research EyeLink 1000+	0.725	0.0240
Tobii X2-60	-	-
Tobii T120	0.158	0.0104
Tobii TX 300	0.171	0.00589
Tobii Spectrum	0.204	0.0454

The Tobii Spectrum and the SMI RED250mobile were tracked with a face mask (Fig. 4). We then started the recording of the eye-tracker using the manufacturer software and/or scripts that record via SDK interfaces, and finally we started the Stepperbox mechanism for rotating artificial eyes using custom-built controlling software.

We then ran each of the staircase, step sequence and continuous sweep scripts, and a subsequent recording of the eyes remaining still.

## Results

### Relationships between precision measures

Figure 8 shows the recording of artificial eye movements from a 1^′^ staircase (the leftmost) to a 10^′^ staircase (the rightmost). It is clear that the movements can be easily distinguished at the 10^′^ staircase, while at the 1^′^ staircase, it is impossible. Somewhere in between, we should find the minimum reliably detectable amplitude. Inspired by the visual inspection method of Crane and Steele (1985), four colleagues (none of the authors) were chosen to be inspectors: all male, age M=46.75, SD = 11.8, with an experience of eye-tracking of between 5 and 21 years and well acquainted with eye-tracker signals. The inspectors were asked to estimate the leftmost staircase, where it is not possible to see the separate step movements in staircases shown in plots like Fig. 8. Each inspector made one assessment per eye-tracker and staircase script. The inspectors scored identically on all eye-trackers, except for the HiSpeed1250, where one inspector reported a value 1^′^ higher than the other three.

Table 4 presents the visually estimated resolution values next to values of RMS-S2S and STD. Surprisingly, the resolution values for most eye-trackers are so good that they could be employed to measure microsaccades, many even with the conservative ceiling amplitude threshold of 15^′^ (0.25^∘^) advocated by Kowler (2011).

**Table 4:** Measurement resolution against RMS-S2S and STD. Resolution values were judged by four experts during visual inspection of plots such as Fig. 8. Precision values were calculated from data where the artificial eyes were still. Here we show the lowest of three values from different intervals

Eye tracker	Resolution	RMS-S2S	STD
DPI Gen5.5	0.025^∘^	0.0069^∘^	0.0292^∘^
SMI HiSpeed 240	0.05^∘^	0.0087^∘^	0.0150^∘^
SMI HiSpeed 1250	0.067^∘^	0.0236^∘^	0.0247^∘^
SMI RED250	0.20^∘^	0.0269^∘^	0.0510^∘^
SMI RED250mobile	0.13^∘^	0.0133^∘^	0.0222^∘^
SMI ETG 2 60 Hz	0.10^∘^	-	-
SR Research EyeLink II (P-CR)	0.025^∘^	0.0071^∘^	0.0292^∘^
SR Research EyeLink 1000+	0.067^∘^	0.0398^∘^	0.0414^∘^
Tobii X2-60	1. 6^∘^	2.879^∘^	1.442^∘^
Tobii T120	0.27^∘^	0.1808^∘^	0.0905^∘^
Tobii TX 300	0.10^∘^	0.3763^∘^	0.2176^∘^
Tobii Spectrum	0.10^∘^	0.1193^∘^	0.0648^∘^

**Fig. 10:**
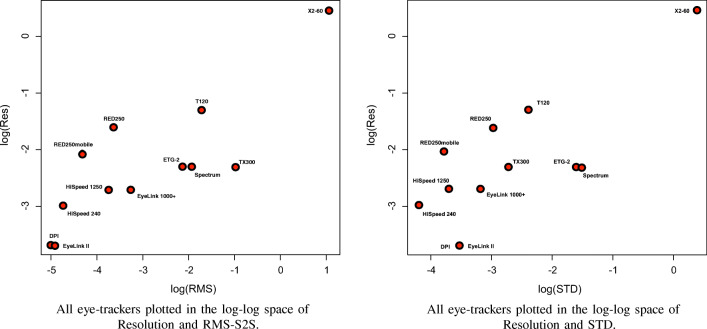
Resolution vs RMS-S2S and STD

Scatterplots revealed that the relations between resolution and precision measures RMS-S2S and STD would be best analysed in log-log space. Scatterplot for the log-log space of resolution vs RMS-S2S (Fig. 10) reveal that the DPI, EyeLink II, and the X2-60 are extremes at each end, and that most eye-trackers are in between. The scatterplot of resolution vs RMS-S2S is very similar to that of STD, with the exception that Tobiis score better on STD and the EyeLink 1000+ and SMIs better on RMS-S2S (a result of filtering), and therefore the order differs.

The somewhat linear relationships seen in Fig. 10 inspired us to calculate the linear regression for the log-log relationship predicting Resolution from RMS-S2S (F(1,10) = 21.03, *p* = 0.001 with *R*^2^ = 0.6681), which leads to:

1 $$ \mathit{\log}\left(\mathrm{Resolution}\right)=0.5\mathit{\log}\left(\mathrm{RMS}-\mathrm{S}2\mathrm{S}\right)-0.7943 $$

This suggests that if the RMS-S2S value is known, a rough estimation can be given of how reliably small movements in the data from an eye-tracker can be seen by an expert. Estimating resolution from STD is also possible but could be less accurate (F(1,10) = 17.15, *p* = 0.003 with *R*^2^ = 0.7473). The relationship, again in log-log space, is

2 $$ \mathit{\log}\left(\mathrm{Resolution}\right)=0.6733\mathit{\log}\left(\mathrm{STD}\right)+0.1540 $$

Excluding the DPI and the X2-60 degrades the results from the linear regressions.

It cannot be denied that the measurement resolution values in Table 4 are low. Generally, noise tends to be reduced in data from artificial eyes compared to data from human eyes. In real recordings with humans, there are several additional factors that can affect the detection of steps. For instance, when we record data with the Stepperbox scripts, we have a full second of absolute stillness between the steps. Such a long inter-step duration provides very good detection conditions that likely make it easier to distinguish the steps and underrate the assessed resolution value. With human data, the periods between microsaccades will be shorter. In addition, periods of stillness will not be very still, as exemplified by the fixation in Fig. 1, because of post-saccadic oscillations, oculomotor drift, and drift because of varying pupil dilation. All this contributes to making detectability of small movements more difficult in human data than in our data, and hence the resolution values in Table 4 should be seen as very optimistic and maybe even unrealistic.

### The extent of the amplitude measurement errors

**Fig. 11:**
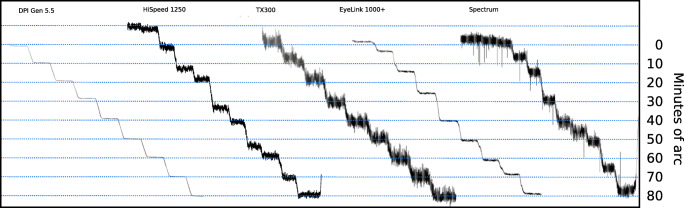
Numbers 3-10 of the ten 20^′^ steps, for five different eye-trackers. Each stop is 1 second long. Blue lines show the actual step size of 20^′^. Notice how recorded step sizes vary in all eye-trackers, and that each eye-tracker has its own specific errors. The data from the DPI have very small errors that could originate from errors in our Stepperbox. The noise in the Spectrum signal is probably because of the artificial eyes, which, however, should not affect step sizes.

**Fig. 12:**
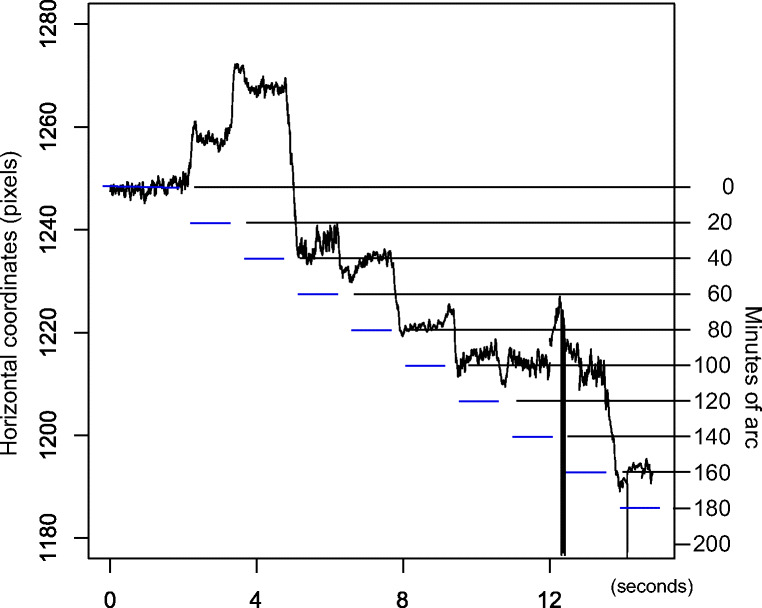
Horizontal gaze for the SMI RED250mobile with artificial eyes making ten 20^′^ leftward movements. Every stop is 1 second long. Blue dashes indicate the nominal Stepperbox movements. The first two leftward steps are recorded as rightward steps, and then a major correction is made in the third step. At around 9000-10000 ms into the graph (x around 1215), steps 7 and 8 are reduced to a zero amplitude.

Figures 11 and 12 reveal that errors are very different between eye-trackers, deviating from the systematic patterns of the Stepperbox. In some places, movements appear compressed in the recorded data, to the extent that sometimes they cannot be discriminated at all. At other places, recorded movements are magnified far beyond the amplitude of the real movement. Some eye-trackers mismeasure amplitudes to the extent that leftward movements are registered as rightward movements. Such direction reversals happen for the SMI RED250mobile all the way up to 20^′^ (Fig. 12), while for most eye-trackers direction reversals only happen below 10^′^.

These incorrectly recorded amplitudes further complicate our search for the correct values for measurement resolution: Fig. 12 exemplifies that many 20^′^ movements are easily detected, as they are much larger than the noise. However, if others of those 20^′^ movements are shrunk to nothing, or even turned into direction reversals, it seems wrong to say that the eye-tracker can reliably measure 20^′^ movements. Because of this interaction between resolution and measurement error, we will therefore spend the rest of the paper quantifying and exemplifying these measurement errors, in order to examine what causes them.

Inspired by and extending Clarke et al. (2002), we decided to calculate the measurement error for steps 3-10 in staircases of each amplitude in the interval 10 − 100^′^ for all eye-trackers except the Tobii X2-60, in which such small steps cannot be distinguished. We measured the distances from the average of each stop to the average at the next stop in the data from staircase scripts, and then subtracted from each such distance the nominal step size of the Stepperbox. Shrunk steps will have a negative error and magnified steps a positive error.

Table 5 summarizes these results, for the amplitude range 10 − 100^′^, where many microsaccades and reading saccades are found. Based on eight movements per amplitude, average error ranges from 2.39^′^ to 23. 5^′^. In percentage of the step amplitude, average errors range from 3.8% to 45%. Those are average errors. In contrast, the worst case errors range from 5.74^′^ to over a degree. In percentage of each of the ten step sizes, the average maximum errors fall between 8% and 79%.

**Table 5:** UAE = unsigned average error, UAE% = Unsigned average error (% of step size), SDerr = STD or errors (equals the resolution measure by Clarke et al. (2002)), AMiE = Average minimum error, AMaE = Average maximum error, while UAMaE% = Unsigned average maximum error (% of step size). All error metrics are calculated for the step range 10 − 100^′^, using data from steps 3-10 for each staircase. For comparison, we also show the Stepperbox laser amplitude errors averaged over 10^′^, 50^′^, and 100^′^ staircases. The averages are calculated on unsigned errors; all other columns are based on both negative and positive error values. Averages are calculated over step sizes, such that the average error percentage reports the typical error as a percentage of the step size, while the average max error reports the average maximum error per step (in arcmin or in percentage of the step size). The Tobii X2-60 is excluded from this table because steps of this size cannot be distinguished with the X2-60

Eye tracker	UAE	UAE%	SDerr	AMiE	AMaE	UAMaE%
Stepperbox Laser	1.56^′^	3.9%	2.09^′^	−4.41^′^	2.20^′^	10%
DPI Gen5.5	2.39^′^	3.8%	3.03^′^	−5.74^′^	9.98^′^	8%
SMI HiSpeed 240	8.76^′^	18.7%	9.93^′^	−22.58^′^	26.29^′^	36%
SMI HiSpeed 1250	6.58^′^	14.2%	8.26^′^	−35.11^′^	30.16^′^	29%
SMI RED250	13.97^′^	22.8%	17.40^′^	−35.35^′^	46.48^′^	48%
SMI RED250mobile	23.49^′^	45.0%	26.74^′^	−66.16^′^	67.64^′^	79%
SMI ETG 2 60 Hz	7.33^′^	10.0%	9.22^′^	−19.10^′^	21.90^′^	28%
SR Research EyeLink II (P-CR)	7.61^′^	17.1%	9.65^′^	−13.58^′^	14.15^′^	33%
SR Research EyeLink 1000+	7.12^′^	15.8%	9.70^′^	−22.83^′^	28.44^′^	34%
Tobii T120	8.27^′^	15.2%	10.52^′^	−35.11^′^	30.17^′^	30%
Tobii TX 300	5.46^′^	12.0%	7.35^′^	−16.11^′^	22.10^′^	27%
Tobii Spectrum	8.18^′^	20.3%	10.53^′^	−20.96^′^	27.80^′^	38%

However, the variation between eye-trackers is large. The DPI has the smallest amplitude errors, near the step errors of the Stepperbox reported above. There is a large midrange group of the high-end video-based eye-trackers with average maximum errors of 30-40%, while the SMI RED250mobile exhibits errors exceeding 1^∘^ for movements that are often smaller than that. In addition, every eye-tracker appears to have its own specific error sequences (e.g. Fig. 11).

Direction reversals, with a negative error larger than the step size, as in Fig. 12, were found in 6.25% of the 80 steps analyzed for the SMI RED250mobile in the 10 − 100^′^ arcmin range, but not for any other eye-trackers in this amplitude range. Errors that shrink the amplitude of the recorded movement more than 50% of the step size were found in all eye-trackers except the DPI and the SR EyeLink II, ranging from 1.25 - 16.25% of all movements for the other systems. Errors that magnify the amplitude by more than 50% of the step size were found in all eye-trackers except the DPI. Large relative errors are more common for small amplitudes: 67.4% of the above large errors were recorded at step amplitudes 10^′^ and 20^′^.

**Fig. 13:**
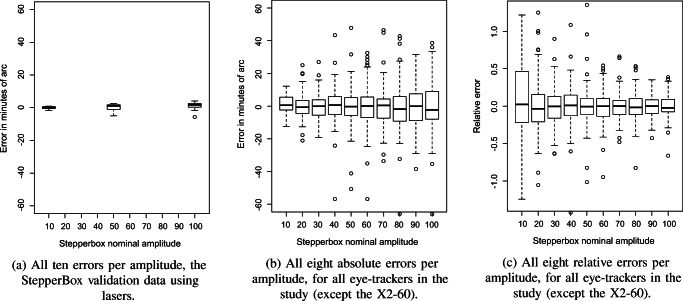
Amplitude errors in the Stepperbox vs eye-trackers. Relative error is defined as measured absolute amplitude error divided by the nominal amplitude. This range covers typical microsaccade amplitudes (Kowler, 2011) and most reading saccades (Rayner, 1998).

Figure 13(b) shows all errors for all eye-trackers next to a comparable boxplot of errors for the laser measurements of the Stepperbox (Fig. 13(a)). This direct comparison visualizes the observation from Table 5 that errors in the eye-trackers are much larger than those of the Stepperbox.

It can further be seen from Fig. 13(b) and (c) that absolute errors in the eye-trackers tend to be larger for larger amplitudes, but relative errors larger for smaller amplitudes. The trend that absolute errors (box plots) become larger with larger step sizes suggests that these amplitude errors continue into steps larger than 100^′^ (1.67^∘^). For the HiSpeed1250, for instance, there were errors of ±0. 5^∘^ for 2. 5^∘^ and 5. 5^∘^ amplitude steps, but errors for all other step sizes were consistently below ±0.33^∘^.

For rotations smaller than 10^′^, which are smaller than the shrinkage and magnification areas (Table 6 below), the gaze direction may remain in a shrinkage area for several consecutive steps. This causes direction reversal to begin to appear for the SMI HiSpeeds, while the EyeLink 1000+ shrinks many rotations with more than 75% of the amplitude, or doubles them in magnification areas (Figs. 8 and 16). Only the EyeLink II in Pupil-only mode and the DPI manage to keep shrinkage and magnification below 50% in this amplitude range, but with real eyes, the EyeLink II would also be susceptible to pupil artefacts, which would add to the errors we report.

**Table 6:** Approximate wavelengths of shrinkage and magnification in the eye-trackers

Eye tracker	Approximate wavelength
DPI Gen5.5	not noticeable
SMI HiSpeed 240	30^′^
SMI HiSpeed 1250	40^′^
SMI RED250	100^′^
SMI RED250mobile	irregular
SMI ETG 2	not noticeable
SR Research EyeLink II	30^′^
SR Research EyeLink 1000+	50^′^
Tobii X2-60	too noisy
Tobii T120	110^′^
Tobii TX 300	100^′^
Tobii Spectrum	120^′^

The exact size of the measurement error seems to depend on how much shrinkage and magnification the recorded data has encountered along the path of the rotation. A majority of shrinkage shortens the recorded rotations relative to the actual amplitude, while a predominance of magnification extends them.

### The prevalence and replicability of amplitude errors

Table 5 shows that for all video-based eye-trackers in this study, at least one of eight recorded rotations has an absolute amplitude error of 30% or more of the movement amplitude. Very few amplitudes are reported without any error.

These amplitude errors appear in data every time we record with the Stepperbox on any of the eye-trackers. We have repeated the measurements on separate SMI HiSpeeds, SMI RED250mobiles, SMI RED250s and Tobii Spectrums in separate labs in South Africa and in Europe. In all these data, we have found very similar amplitude errors every time.

In addition, we recorded staircase scripts and step sequences for all combinations of number of calibration points (representing different polynomials) and filter settings on the SMI HiSpeed240 and the RED250mobile. The same amplitude errors were seen in all data, every time.

A colleague at SR Research, who built his own monocular Stepperbox, offered to record staircase data with his machine, his artificial eyes, and his EyeLink 1000+. Those data exhibited very similar errors, amplitude mismeasurements, that we saw in our data^2^.

During February 2019, the authors let Tobii Pro in Stockholm borrow our Stepperbox and test their Spectrum eye-trackers in-house. The resulting data exhibited very similar amplitude mismeasurements to those presented here.

Figure 8 above shows how errors in the gaze signal do not reflect the order of the steps, but the horizontal position on the monitor toward which the eye is directed. In data where we let an artificial eye make a back-and-forth sweep or step sequence, the same amplitude errors repeat every time the eye points at the same horizontal coordinates.

This ubiquitous prevalence of errors reflects the fact that eye-trackers are machines which are built to be reliable in exactly this sense: They produce the same measurement, whether correct or erroneous, again and again.

### Amplitude errors and the different resolution measures

**Fig. 14:**
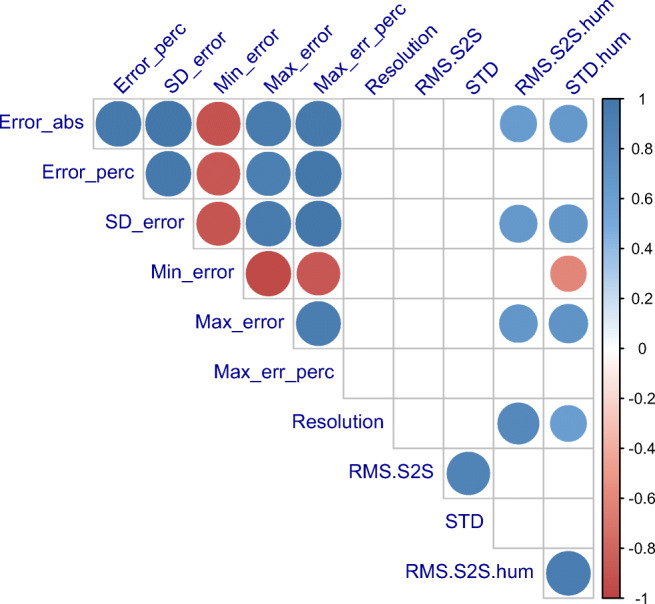
Correlation matrix for the measures reported in Tables 4 and 5. Only correlations with a significance better than 0.05 are shown in this matrix. A colour key is shown on the right. The minimum error correlates negatively with everything; all other correlations are positive. Resolution, the minimal reliably detectable movement amplitude, does not correlate with any of the error-metrics, nor with SD error, the metric called “resolution” by Clarke et al. (2002). Neither does resolution correlate with RMS-S2S and STD of artificial eye data. However, resolution correlates with both measures of precision for human data (taken from Holmqvist (2015) and manufacturer spec sheets). All measures of amplitude errors correlate strongly within their group (upper left), and only in part with the human precision measures.

We then investigated the relation between the measurement errors in Table 5 and the resolution defined by experts in Table 4 as the minimum reliably detectable movement. It is clear from the correlation matrix in Fig. 14 that there are no significant correlations between resolution and the six error measures. Not even the SD of the error, defined by Clarke et al. (2002) as resolution, correlates with the other resolution. This suggests that measurement errors and the smallest reliably detectable movement are two unrelated properties of the eye-trackers.

However, Fig. 14 confirms the previous finding in Fig. 10 that the smallest detectable movement is related to the level of noise in the data, albeit more strongly in human data.

### Wavelengths of the cycles of shrinkage and magnification

Inspection of plots from Stepperbox data such as Fig. 8 reveals that shrinkage and magnification of step amplitudes tend to replicate at specific gaze directions. The CR signal from smooth rotations also exhibit a regular, repetitive pattern (Fig. 7). We calculated the interval of replication (the wavelength) of these cyclic repetitions by measuring the distance between two occurrences of maximum shrinkage in five different parts of the data, and rounding the average to the closest ten arcmin. Table 6 summarises these results.

Some eye-trackers have a wavelength of half a degree, while other systems exhibit two degree distances between shrinkage maxima, three times larger than others. The DPI and the SMI ETG 2 had no noticeable alternations, and the SMI RED250mobile has very irregular errors.

## What is the origin of the measurement errors?

The existence of the measurement errors in Table 5 by necessity implies that many recorded eye movements are either compressed or extended, compared to the real amplitude. Because we do not have a complete understanding of the interior operations of each eye-tracker, we cannot list every possible explanation of shrinkage and magnification and the resulting mismeasurements. A few explanations can be examined within our current data set, though.

### Gaze position, step order, or movement direction?

The shrinkage and magnification zones in Fig. 8 suggest that the specific errors in data from the EyeLink 1000+ are a function of gaze position and not of step order. In order to show that gaze position also predicts errors across the entire monitor space for other eye-trackers, we took the gaze data from the artificial eyes captured during the back-and-forth homing movements. Since the Stepperbox makes smooth sweeps, the gaze data should be smooth as well.

**Fig. 15:**
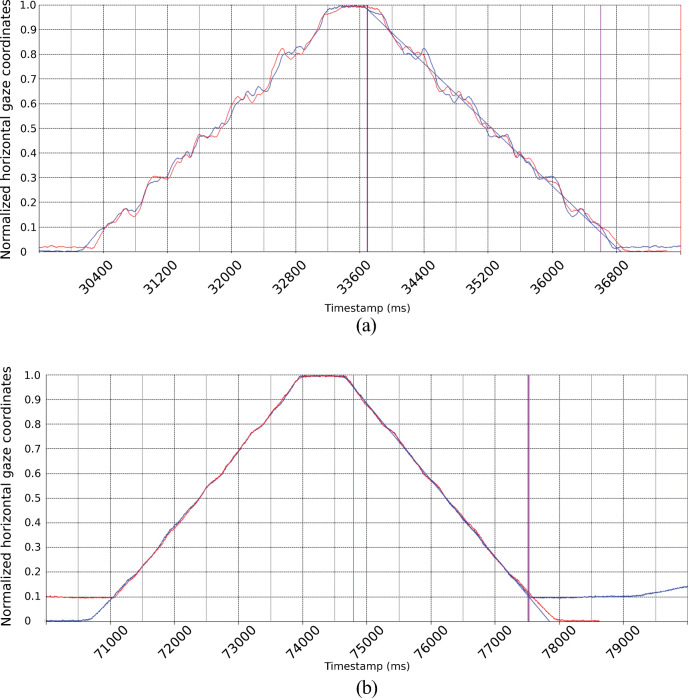
Data from a smooth homing movement (blue), right to left and back, has been mirrored and overlaid (red) onto the original signal. The close matches between rightward and leftward signals reveal that the specific errors are a function of gaze direction but not of movement direction. A regression line shows the estimated smooth movement. The RED250 exhibits much larger deviations from the line.

Figure 15(a) shows data for the SMI RED250mobile, which are clearly not smooth. The blue signal shows the original gaze data, while the red signal is the reverse of the original. When we overlay the original and its reverse, the match is very close, suggesting that on repeated sweeps, errors repeat at the same gaze position throughout the measurement space.

In order to quantify how closely the original and the reverse signals matched, we first found the best match by adjusting the amount of shift to minimise the sum of distances between the x-coordinates of the original and reversed signals, over all samples within the interval. We then calculated fit as the standard deviation of these differences. In order to compare different eye trackers, the x-coordinates were scaled to fit in the range 0 to 1.

The best fit of x-differences between the original and reversed signals for the data in Fig. 15(a) gives an average sum that is very close to zero (0.01), but the standard deviation of the differences (0.0129) is much larger for the SMI RED250mobile than for that of the EyeLink 1000+ (Right eye: 0.0012) in Fig. 15(b). This indicates that the mismeasurements of the RED250mobile occur at approximately, but not always exactly, the same position on the screen, while the EyeLink 1000+ reproduces errors like a clock.

Table 7 quantifies the similarity we see in Fig. 15 over a range that excludes the turning point and the transition to the other cycles. The lower the standard deviation of differences, the more similar are the data from repeated smooth scans, including the errors. The EyeLinks have the most repeatable errors, while the errors in the remotes repeat very regularly but with some small differences (illustrated in Fig. 15).

**Table 7:** The differences between repeated smooth scans across the monitor provide a measure of the degree of replicability of these errors. The unit is pixels

Eye tracker/eye	Diff/sample (px)	STD of diff
EyeLink 1000+ Left	-0.20	0.0062
EyeLink 1000+ Right	-0.25	0.0012
EyeLink II Left	-0.78	0.0051
EyeLink II Right	0.24	0.0051
TX300 Left	0.25	0.0149
TX300 Right	-0.02	0.0307
RED250 Left	0.01	0.0129
RED250mobile Left	0.01	0.0232

### Calibration mathematics?

The observation that the errors repeat at the same gaze direction suggests that it is conceivable that higher order calibration polynomials could have ripples (alternating maximum and minimum points) in the measurement space that locally shift data in the horizontal direction. Calibrating with a pair of human eyes for a recording made with artificial eyes could conceivably make this worse.

There are four reasons why calibration routines are not a plausible explanation of these errors. Firstly, as Table 3 shows, the mapping produced by the calibration varies very little across the monitor space for each of the eye-trackers.

Second, if calibration is the cause of the errors in Gaze, because of the polynomial calculations taking correct Pupil and CR signals as input, why do we find repetitive errors only in the CR (Fig. 7)?

An additional argument against calibration polynomials contributing to the errors would be that the TX300 and the Spectrum reportedly do not use polynomial calibration but model-based gaze estimation, and these eye-trackers are still as much affected as the SMIs and the EyeLink, which use polynomials.

Finally, the polynomial order of existing calibration algorithms is too low to explain the errors. In the investigations of Blignaut (2014) and Cerrolaza, Villanueva, and Cabeza (2012), the maximum polynomial order of the best polynomials for calibration is of the order of 4 and 5. Because a 5th order polynomial has 4 turning points, polynomials of that order could only have caused four switches between shrinkage and magnification. However, the period of the shrink/magnify alternation that we find in data is often short, down to 30^′^, which would imply at least 40 errors over a calibration space of 20^∘^, not 4-5 as predicted by the hypothesis that calibration polynomials are the cause.

There is a remote possibility that manufacturers use an initial polynomial to get a rough estimate of gaze coordinates. Then based on the initial estimation, they can select the three/four/five nearest calibration points and recalculate the mapping polynomial. This would mean that it is possible to have many different polynomials, each one with two/three/four turning points. This is done in Blignaut, Holmqvist, Nyström, and Dewhurs (2014), but we do not hold it likely that this is implemented in commercial eye-trackers.

All this makes it improbable that polynomial calibration functions cause the errors.

### The corneal reflection vs the pupil?

Figures 6 and 7 above suggest that the issue may stem from the CR or, more accurately, the centre of the image in the eye camera of the reflection of infrared light in the cornea. The CR has been considered a better signal than the pupil (Hooge et al., 2016), likely due to its frequent use in many eye-trackers from 1901 to today (Holmqvist & Andersson, 2017, p. 67-70). However, as we saw in Figs. 6 and 7, the CR does not move with the Stepperbox laser the way the Pupil does, nor as smoothly as the Pupil when the artificial eyes are rotated smoothly.

The Pupil signal, more accurately, the centre of the pupil image in the eye camera, has been blamed for several amplitude and gaze position artefacts in eye-movement data resulting from dynamics in the human pupil itself (Hooge et al. 2019; Hooge et al. 2016; Holmqvist 2015; McCamy et al. 2015; Drewes 2014; Drewes, Masson, & Montagnini 2012). However, in our measurements with a pair of artificial eyes, the pupils are static, physical features that will neither oscillate, constrict nor dilate, and therefore none of those pupil-induced artefacts can happen in our data.

Otero-Millan, Castro, Macknik, and Martinez-Conde (2014) have suggested that “the corneal reflection coming in and out of the pupil can introduce a quick change in the position of the center of mass of the pupil”. However, in a longer horizontal sequence, pupil border passages would happen at most twice, while Fig. 7 above shows a large number of errors in the CR signal along the path of a smooth sequence. Similarly, the CR size and center changes considerably during passage of the limbus, but again, limbus passages happen rarely.

We will now examine whether the errors in the Gaze signal stem from errors in Pupil and CR signals. Pupil and CR position in the eye camera are available in the data from most SMI and EyeLink trackers, and hence all following analyses are made on those systems. Tobii does not supply Pupil and CR position data, but we have no reason to believe that the internal Pupil and CR signals in Tobii eye-trackers would behave any differently.

#### Are errors of the CR larger than errors of Pupil?

Figure 6 above suggests that the error in the CR is larger than the error in the Pupil signal. In order to examine whether this is generally the case, we quantified errors in Gaze, Pupil, and CR signals from high-end eye-trackers that provide these data, after calibrating each signal separately. Table 8 shows that, almost always, the errors in the CR are indeed larger than the errors in the Pupil. Because the Gaze signal is determined by Pupil and CR, the implication would be that the CR is the larger contributor to errors in Gaze.

**Table 8:** Top: The EyeLink 1000+ with 50 mm vs 35 mm lens. No clear benefits can be seen of the 50 mm lens. Bottom, for comparison: The SMI HiSpeeds. Data from 10, 50 and 100^′^ staircases (not 10-100^′^ as Table 5), steps 3-10. Note how with very few exceptions, irrespective how we measure, the error in the CR is larger than the error in the Pupil signal. Similarly, the EyeLink II (middle) is somewhat better in pupil-only mode compared to P-CR mode (also see Fig. 16)

Eye tracker	UAE	UAE%	SDerr	AMiE	AMaE	UAMaE%
EyeLink 1000+ 50 mm lens						
Gaze	5.10^′^	11.1%	8.36^′^	−6.16^′^	10.69^′^	35.8%
Pupil	2.08^′^	5.77%	2.82^′^	−2.96^′^	4.20^′^	10.0%
CR	4.69^′^	10.3%	4.87^′^	−5.51^′^	16.45^′^	22.2%
EyeLink 1000+ 35 mm lens						
Gaze	5.06^′^	14.5%	7.24^′^	−6.87^′^	10.34^′^	27%
Pupil	2.19^′^	6.0%	2.67^′^	−3.09^′^	3.84^′^	8.6%
CR	4.54^′^	8.9%	5.29^′^	−8.13^′^	7.81^′^	15.1%
EyeLink II						
P-CR (Gaze)	5.91^′^	18.4%	7.91^′^	−11. 0^′^	12.73^′^	32.9%
Pupil-only	4.76^′^	10.1%	6.31^′^	−9.53^′^	9.48^′^	17.7%
SMI HiSpeed 1250						
Gaze	6.19^′^	17.7%	7.54^′^	−6.62^′^	12. 1^′^	22.3%
Pupil	3.23^′^	8.1%	3.69^′^	−1.19^′^	7.63^′^	11.4%
CR	5.30^′^	7.9%	5.30^′^	−5.15^′^	11.46^′^	10.9%
SMI HiSpeed 240						
Gaze	11.94^′^	29.4%	14.01^′^	−8.92^′^	17. 6^′^	40.3%
Pupil	5.76^′^	4.7%	5.14^′^	−4.25^′^	10. 2^′^	13.8%
CR	6.65^′^	15.6%	8.03^′^	−8.86^′^	20. 2^′^	14.7%

**Fig. 16:**
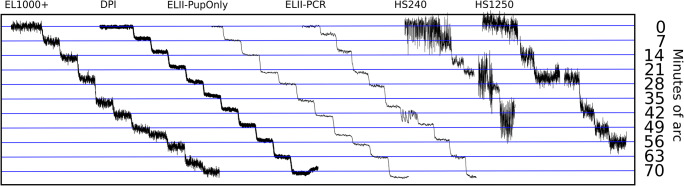
7^′^ staircases for six high-end eye-trackers. Each stop is 1 second long. At this step amplitude, the EyeLink1000+ significantly mismeasures the amplitudes of most steps, while in comparison, the DPI exhibits very small errors. Remarkably, the noise in the EyeLink II in Pupil-Only mode is lower even than in the DPI, but the EyeLinkII mismeasures several of the amplitudes. In P-CR mode, the EyeLinkII has larger errors than in Pupil-only mode. The two SMI systems are so heavily affected by shrinkage and magnification that very few of the step amplitudes are correctly measured.

The EyeLink II records in either P-CR or in pupil-only mode. If the CR would be the major contributor to the error, we would expect smaller errors in the pupil-only mode, which Table 8 confirms.

**Fig. 17:**
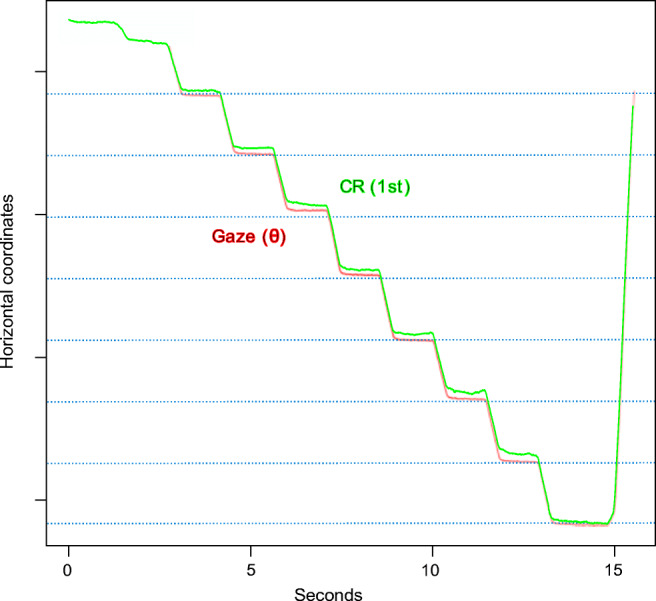
Overlays of CR and Gaze of the DPI Gen5.5 during 20^′^ staircase steps. Every stop is 1 second. Gaze (red) moves in more accurate steps than CR (green).

The DPI also tracks the CR (the 1st in DPI terminology), and in Fig. 17, we have plotted the DPI CR next to the DPI gaze for 20^′^ staircase steps. The CR is marginally more erroneous than gaze, but the errors in both signals are much smaller than for VOGs.

#### Increasing the number of pixels for the CR by changing the lens of the eye camera

It is possible that in modern digital camera systems the CR may cover very few pixels. In SMI systems and the EyeLink, the pupil diameter often spans around 10-100 pixels in the eye camera while the CR diameter spans 2-15, as measured by pupil diameter and CR area values in the data files, and counted in available images of the eye. The movement of the CR in the eye camera can be as small as 0.06 camera pixels for a 10^′^ eye movement (Fig. 9) when the CR diameter is around 8 pixels. The algorithms used by manufacturers in estimating subpixel movements are not published, but they may make the CR more susceptible to quantization errors than the Pupil signal during small movements, which could explain the behaviour of the CR signal. In support of this, we note that the SMI RED250 and RED250mobile, with the largest errors in Table 5, have the smallest pupil diameters in our data set (16 and 10 camera pixels, respectively), while the SMI ETG with comparatively small errors has the largest pupil diameter (117 pixels).

SR Research recommends that their customers record microsaccade data with a 50 mm lens rather than with the standard 35 mm lens we have used on the EyeLink when recording all data presented above. With the 50 mm lens, more of the camera pixels are used for the pupil and corneal reflection images, and fewer pixels are busy recording empty space. It should be noted that we have not found any publications mentioning the use of the 50 mm lens, so it is possible that the recommendation by SR Research is not that widely followed by their customers.

The change of lenses did have an effect on the sizes of pupil and CR images. The horizontal diameters of the L / R pupil were 34 / 37 pixels with the 35 mm lens and 54 / 58 with the 50 mm lens. The CR diameters were 8.7 and 7.6 pixels with the 35 mm lens, but 14.2 and 12.2 pixels with the 50 mm lens. However, as Table 8 illustrates, we could observe but a very modest reduction of amplitude errors with the 50 mm lens.

This result supports the conclusion that although the CR is a major contributor to the observed errors, quantization in the eye camera is not the cause, at least not for the EyeLink1000+. Replicating this test on more eye-trackers would be desirable, but it is not clear how to do it. We considered replicating this test on the EyeLink II by moving its camera arm closer to or further away from the artificial eye, but the EyeLink II illumination is located on the same arm as the camera, so this modification would introduce a variation in light levels that would not make this a well-controlled test of resolution.

### Errors as disagreement between Pupil and CR signals

The fact that errors are generally larger in the CR than in the Pupil (Table 8) must be seen from the perspective that in P-CR eye-trackers, the Pupil and CR jointly combine to form the Gaze signal, once per sample. That is simply how they are built to work. Although the difference in averages tell us that the CR contributes more to the errors, averaging hides the fact that the minute interaction at each point in time between the two features from the camera sensor is what really matters.

**Fig. 18:**
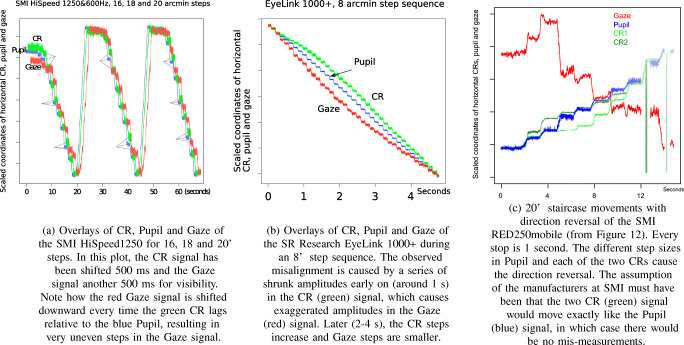
Examples of how the interaction between CR (green) and Pupil (blue) errors results in errors in Gaze (red). The data have been scaled so that when steps are regular, blue and green, and in (a) and (b), also red, would coincide. In all three examples, the CR contributes more to errors than the pupil.

In Fig. 18(a), we plot CR, Pupil and Gaze signals from a 16-20^′^ staircase recording from the HiSpeed 1250. Detailed inspection makes it clear that misalignment of Pupil and CR signals coincides with, causes, a larger mismeasurement of Gaze. All stops with mismatches have been marked in Figure 18(a). In all non-marked stops, Pupil, CR and Gaze coincide.

To confirm that large errors in the CR also contribute to errors in Gaze in the EyeLink1000+, we have plotted the CR, Pupil and Gaze signals from an 8^′^ step sequence with the EyeLink1000+ (Fig. 18(b)). We can notice the same happening in the EyeLink as in the SMI HiSpeed: in the first second of data, shrunk CR movements lead to magnified gaze movements. After 2-3 s, CR movements are larger, and Gaze steps smaller. Figure 18(b) also shows how a series of amplitude mismeasurements can be a contributing factor to non-linearity: the non-linear data are seen in the curvature of the Gaze signal. This non-linearity causes a small offset, which, at its peak in Fig. 18(b), is about half a degree.

**Fig. 19:**
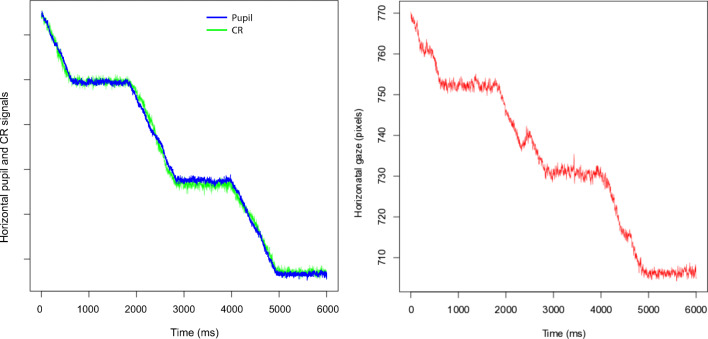
On the left side, Pupil and CR signals from 50^′^ steps recorded with the SMI HiSpeed 1250. On the right side, the resulting Gaze. Every step is 1 second long. In the rotation between the two first stops, the Pupil signal slows down and the CR speeds up. In Gaze, we can observe a simultaneous artefactual backward movement.

Not only amplitudes of movements are affected by the errors in the CR (and pupil) signals. False events in data appear to be generated by small variations in the relative positions of Pupil and CR signals. Figure 19 shows how a slight shift of CR and Pupil coincides with a backward-moving artefact. The same pattern can be distinguished in the preceding and subsequent steps.

### Eye-trackers with two or more corneal reflections

Many eye-trackers have dual or even more CRs per eye, and if the two CRs of a single eye move independently and differently, each CR may pull gaze in a different direction.

For instance, in Fig. 18(c), we plot the two CRs, the Pupil and the Gaze signal for the 20^′^ movements with direction reversal we saw in Fig. 12. Close inspection of the relative movement of CRs and Pupil signals reveals that when Gaze amplitudes are mismeasured, the motion in one of the two CR signals is larger than the motion in the pupil signal. When the direction reversal in Gaze happens, in Steps 1 and 2, the motion in the CR2 is larger than in the Gaze signal, while the CR1 moves with Gaze. In the third step, the large correction, the motion in the Pupil signal is large and both CRs exhibit a small movement only. For the three following and reasonably correct gaze amplitudes, the CR and Pupil signals are of the same size. For the two zero-amplitude movements in gaze, the CR1 signal changes more than the Pupil and CR2 signals. It is very likely that this difference in the motion between the two CRs *caused* the direction reversal as well as the zero amplitude for two steps. The reported pupil diameter is 15.5 camera pixels for this recording, which does not leave many pixels for the CRs).

We do not know how the two CRs are mathematically combined with the Pupil signal to produce the Gaze coordinate in the SMI RED250mobile, but data show a complex dynamics resulting from the two CR signals not moving together. Most remote eye-trackers today have two corneal reflections. Unfortunately, Tobii systems do not report the movements of Pupil and CR signals in their data files, so we have not been able to investigate their data in detail, but given that the gaze data from Tobiis exhibit the same artefacts, we have no reason to believe that their dual CR signals will behave differently from dual CR signals in SMI trackers.

In contrast, the SMI ETG have six corneal reflections per eye. Interestingly, Table 5 revealed that the errors in step amplitudes are lower for the SMI ETG than for many other VOGs. It is possible that the six CRs combine to outweigh or average the effect of errors in single CRs, thereby creating a more stable Gaze signal. Unfortunately, however, we cannot inspect how these six CRs behave, because the output of the SMI ETG data does not include CR signal data.

### Replication on six human saccades

**Fig. 20:**
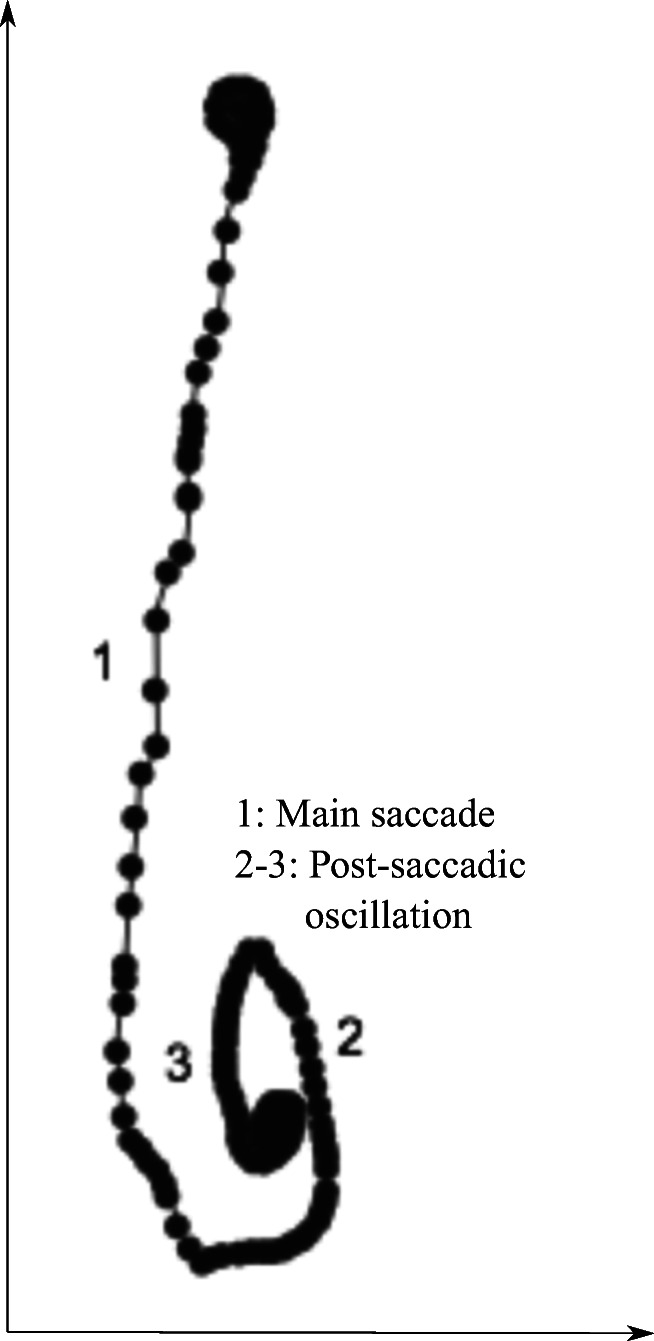
Irregular sample-to-sample distances (point 1) in a human vertical saccade, with a sizable post-saccadic oscillation (2-3). Recorded with the SMI HiSpeed 1250, and reproduced from Holmqvist and Andersson (2017, p. 161).

Our final example attempts to replicate, in human data from the SMI HiSpeed 1250, the errors resulting from shrinkage and magnification. We did not want to let people make staircases or step sequences of 20^′^ saccades, because the errors in the recorded data would consist of a combination of errors from the oculomotor system, errors due to the human pupil, and errors because of the CR. However, there is another way to replicate on humans. Previously, Holmqvist and Andersson (2017, pp 153 and 161) showed plots of individual gaze samples during saccades with irregular sample-to-sample distances (see also Fig. 20). This variation could be due to temporal imprecision of the eye-tracker, but the standard deviation of intervals between samples in these HiSpeed data is 0.0029 ms, for 2 ms intervals at 500 Hz, which makes temporal imprecision an unlikely explanation of large variations in sample-to-sample distances in human saccades.

An alternative explanation to irregular spatial sampling, in line with results presented above, would be that these irregular sample distances in human saccade data result from the same alternating shrinkage and magnification. Our hypothesis would be that when the saccade passes through shrinkage and magnification areas, the sample-to-sample distances are alternatively shortened and prolonged, which should be seen as a jerky signal when we plot it. We decided to investigate human 16^∘^ horizontal saccades at 500 Hz on the SMI HiSpeed 1250. A 16^∘^ saccade is likely to pass through many periods of magnification and shrinkage near its peak velocity at an estimated 500^∘^/s. Notably, the sample-to-sample distances near that velocity peak would correspond to around 1^∘^ (60^′^), which is in the interval we have investigated in Table 5.

**Fig. 21:**
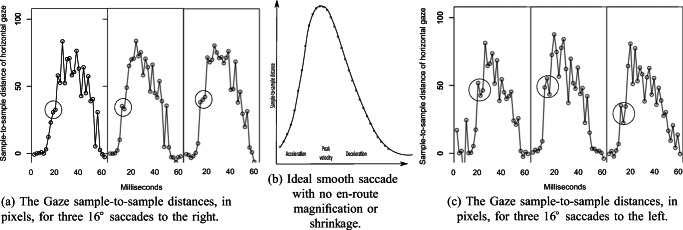
In (a) and (c), sample-to-sample irregularities in human saccades. The alternating shorter and longer distances are consistent with shrinkage and magnification. Circles mark the effect of what could be strong shrinkage areas, embedded in magnification areas, repeating across the three saccades of each direction. In (b), the expected sample-to-sample distances in an ideal saccade with no shrinkage or magnification.

Figure 21 shows these saccade sample-to-sample distances for six 16-degree human saccades from the SMI HiSpeed 1250. Irregular sampling is clearly seen in the gaze signal, in the form of wildly alternating sample-to-sample distances that do not represent the smooth acceleration and deceleration that we expect of saccades. On the accelation side of the saccades, we have marked instances of shrinkage effects with circles. These results are consistent with magnification and shrinkage happening also in human eye movements.

## Discussion and outlook

Our study has asked questions rarely asked before, using new methods. In sum, it appears that eye-trackers are reasonably good at *detecting* small rotations of artificial eyes (Table 4), but also that they often *mismeasure* the amplitudes of those rotations, both with artificial and human eyes (Table 5 and Fig. 21).

These results address two assumptions that apparently must be rejected: firstly, that the reported amplitudes of small movements measured with video-based eye-trackers can be taken at face value, and secondly, that the CR signal from video-based eye-trackers is a reliable signal.

We have also shown that the minimum reliably detectable eye-movement (resolution according to Poletti & Rucci (2016) and Holmqvist & Andersson (2017)) is unrelated to the standard deviation of the measurement error (resolution according to Clarke et al. (2002)), and that precision measured by RMS-S2S or STD reasonably well predicts the minimum reliably detectable movement, at least with artificial eye data.

### The method of rotating artificial eyes

This study was done using a mechanism for rotating artificial eyes. Artificial eye recordings are our only way to systematically control the input amplitude (Reingold, 2014, e.g.). The Stepperbox is a low-cost solution (below 2000 €) with certain drawbacks. Firstly, slack reduces the amplitude of the first few movements. Slack can be solved by building in slack-consuming movements before the measured rotations. As a further precaution, we analysed only amplitude errors in staircase movements number 3-8 in the staircase patterns.

Secondly, our laser-pointer validation shows that there is a small but systematic error in the remaining steps, but fortunately, the errors in most eye-trackers are much larger (Fig. 13). Our validation above shows that our Stepperbox produces rotations that systematically deviate from the nominal rotation by 4% on average, far below the much more variable amplitude errors measured in the VOGs (10-45%), but interestingly of the same size as the errors we measured in the DPI. In other words, a simple Stepperbox appears to be good enough also for an in-depth study of how small movements are registered in eye-trackers.

For studies of very minute errors in eye-trackers, there are other solutions. For instance, the high-end rotating mechanism for artificial eyes used by Clarke et al. (2002) was built at a cost of 30000 €^3^. Another option would be to build a piezo-electronic rotating machine, but to our knowledge no such machine has been used to investigate data quality of eye-trackers. In principle, it would also be possible to play a video of eyes with very controlled movements in front of the eye-trackers, but that video would have to include a CR which is not a reflection of the illumination in the eye-tracker, which makes it a less complete test method, and may introduce new artefacts.

What is the evidence that these results translate to human data? It is impossible to replicate these exact findings with human data, because although humans can make small saccades in the microsaccade range, we cannot expect of humans to make sequences of a specific amplitude to within 4% error, and even if we could, how would we validate those sequences? However, we can let humans saccade over the putative shrinkage and magnification zones and notice uneven sampling along the saccade, which would be consistent with such zones, as shown in Fig. 21.

Note that rotations of artificial eyes give eye-trackers optimal recording conditions. Rotations of human eyes add many additional issues that would add to the errors we have shown. When pupil errors (Drewes, 2014) add to CR errors, or we add more noise (Holmqvist et al., 2012), small head movements (Niehorster, Cornelissen, Holmqvist, Hooge, & Hessels 2017), or optical artefacts such as additional IR light, or tears (Holmqvist & Andersson, 2017, p. 140-141), then the different errors can be expected to add to one another. Add to that imperfect event detection, and the measurement of small human saccades with VOG eye-trackers comes across as a perilous activity.

### Resolution values and the variable errors of movement amplitude measurements

We set out to measure a single variety of measurement resolution, the *smallest movement that can be reliably detected* by a human observer, inspired foremost by Poletti and Rucci (2016) and Clarke et al. (2002). We could indeed find measurement resolution values that follow this definition (Table 4) and show that these resolution values can be predicted from RMS-S2S and STD values of the same eye-trackers (Fig. 10), and also correlate significantly with other precision data from humans (Fig. 14). This finding quantifies our intuitive expectation that increasing noise drowns out ever larger movements.

But noise alone only partly determines the practical spatial measurement resolution of an eye-tracker. A movement of a small amplitude is easier to detect if embedded between two long periods of absolute stillness. If instead the small-amplitude movement is embedded in drift and post-saccadic oscillations (PSOs), or very short inter-movement intervals, the movement is more difficult to detect, even in a recording with low levels of noise. Add to that the fact that precision (RMS-S2S and STD) varies dramatically between participants, depending on individual factors in physiology and eye wear (Holmqvist, 2015). All this makes measurement resolution dependent on all these factors, and the ideal resolution values we presented in Table 4 should not be taken to indicate what size of human eye-movements can be measured.

Equally important is that the *reliability* component in the definition of measurement resolution forces us to question a straightforward relationship between noise and resolution. Our data show that all commercial VOGs (video-based eye-trackers) exhibit measurement errors that vary in size across the measurement space. It has the curious consequence that a 10^′^ microsaccade can be reported as 10^′^, but if the microsaccade had been produced a tiny bit to the left or to the right, it could also have been recorded with a 3^′^ or a 25^′^ amplitude. That tiny bit between shrinkage and magnification area is often less than a degree in the measurement space, but could also be a few millimetres translation (sideways movement) of the participant at a 70 cm viewing distance.

When small saccades are seriously mismeasured, it undermines the very definition of measurement resolution as we understood it in the beginning of this paper, because how can we say that a 30^′^ movement is reliably measured if it is recorded in data as having an entirely different amplitude?

The alternative resolution measure by Clarke et al. (2002) quantifies the size of such measurement errors. Figure 14 shows that Clarke et al’s measure does not correlate with the amplitude of the minimum reliably detectable movement. This is expected as there is no reason to suspect that noise and amplitude errors are related. Rather, these two measures of resolution appear to describe two largely unrelated aspects of the signal.

Although measurement errors (Tables 5 and 8) were computationally calculated, we have found the smallest detectable movement using human visual inspection of plots (Table 4). Algorithmic detection would have been preferred, but which algorithm? All event detectors make assumptions about data, built in by their designers. Also, in recent time, the best event detectors are built on human coding of data (Zemblys, Niehorster, Komogortsev, & Holmqvist 2018b, e.g). Expert human coding is not perfect (Hooge, Niehorster, Nyström, Andersson, & Hessels 2018), but is usually on par with or better than the most recent algorithms (Zemblys, Niehorster, & Holmqvist 2018a, e.g.). Human expert detection of the smallest discernible movement seems like the most reasonable choice.

### Consequences of erroneous amplitude measurements in VOGs

Video-based eye-trackers have been the measurement tools of choice for more than 25 years. Assuming our data are correct and replicable, branches of science where small saccades need to be measured with any of the eye-tracker we have tested will likely have been affected to some degree, for instance research on micro-saccades, reading research and research on vergence as three examples.

*Microsaccade studies that report amplitude or velocity* from video-based eye-trackers: Google Scholar reports that 87 papers to date report microsaccade amplitudes and 28 papers microsaccade velocity^4^, with data mostly from the EyeLink II. Our data show that precision is better (the value is lower) in the EyeLink II than in EyeLink1000+, which researchers can see from data plots. Possibly they therefore preferred the EyeLink II over other eye-trackers for microsaccade research. In contrast, amplitude mismeasurements are very difficult to see in data from humans, and appear to be unrelated to precision and resolution, and so this property (amplitude mismeasurements) of eye-trackers was likely never taken into consideration, neither by researchers choosing equipment nor by manufacturers developing it.

It is impossible to say whether the amplitudes and velocities reported in microsaccade papers based on EyeLink data are correct or not, as it depends on whether the microsaccades were executed in a part of the measurement space which is neutral, which shrinks or magnifies, during the authors’ particular data collection. Given that very small movements of the eye (or head) are needed to take gaze between shrinkage and magnification, most publications on microsaccades are likely to have collected a mixed bag of shrunk, magnified and correctly measured microsaccades.

Indirectly, our results also question detection algorithms for microsaccades that rely on amplitude or velocity. Such algorithms will of course detect movements in the data file, but many of these movements will not be correctly measured microsaccades. Algorithms that directly or indirectly rely on the corneal reflection can have the same issues. These algorithms will discount correct microsaccades as being either too long or too short, and the number of correctly detected microsaccades will be too small, while some small movements that should be under the threshold will be magnified to look like qualified microsaccades.

The presence of monocular microsaccades in P-CR eye-tracking has been accepted by some researchers, while others have suspected it to be an artefact. Recording with a binocular DPI and coils, Fang, Gill, Poletti, and Rucci (2018) found only binocular microsaccades, while researchers who have used P-CR video-based eye-trackers often have reported monocular microsaccades (e.g Engbert & Kliegl (2003); Gautier, Bedell, Siderov, & Waugh (2016)). Fang et al. (2018) point out that the (mis-)detection of monocular microsaccades in P-CR video-based eye-trackers results from a complex interplay of factors such as noise and settings in detection algorithms. Our results present an additional explanation of how this could happen. It is very conceivable that binocular microsaccades on the border of the amplitude threshold were sometimes shrunk in the data from one eye (to below the threshold) and magnified in the other eye (to above the threshold), which would artificially result in what appears to be a monocular microsaccade.

Interestingly, there has been a considerable shift upwards in amplitude thresholds for microsaccades, from around 5 − 10^′^ (appr 0.1^∘^) (Ditchburn & Ginsborg, 1952, e.g) to more than 1.5^∘^ (Martinez-Conde, Macknik, Troncoso, & Dyar 2006, e.g). Size matters crucially to the microsaccade definition, as a 1.5^∘^ saccade moves the fovea 10 times more than the microsaccades measured in the 1950s and 1960s. This difference is very likely to have perceptual consequences, so it is not surprising that Kowler (2011) argues that movements larger than 15^′^ should not be considered microsaccades, but ordinary saccades. Interestingly, we could show that many of the current VOG eye-trackers can in fact *detect* some movements below 15^′^ under ideal conditions, although they cannot correctly *measure* the amplitude of any movements in the microsaccade interval. It is possible that when, after the switch to VOGs, some small movements were shrunk and some magnified to an amplitude above the old detection threshold, it affected the distribution such that the amplitude criterion had to be increased to allow for the same amount of microsaccades to be detected.

In *research on reading*, we find the vast majority of saccades in the amplitude range 0.5 − 3^∘^ (Rayner, 1998). Measured amplitudes in this range are likely to have errors up to around 0. 5^∘^ (or 50% of typical 1^∘^ saccade during reading), which in turn affects landing positions, skip rates and other position measures employed in reading research. A simple Google Scholar search reveals that over 1000 published papers report saccade amplitudes during reading with data from the EyeLink alone, and a similar number for data from SMI eye-trackers. Again, the validity depends on the minute particularities in the recording situation.

VOG eye-trackers are sometimes used in research on *vergence*. Very recently, Wang, Holmqvist, and Alexa (2019) and Hooge et al. (2019) independently showed that noise and small inaccuracies in binocular (x,y) gaze, caused by pupil dynamics, translate to very large errors in the (z) depth dimension. It is a fair guess that the artefacts from the CR will add to the errors they report.

In saccades with larger amplitudes, such as our 16^∘^ saccade above, the many errors along the saccade path should roughly even out. The total error will play but a small role for the large saccade amplitude recorded in the eye-tracker. At this amplitude the problem is another: the uneven sampling leaves behind it a signal that looks noisy, but that noise is different in origin from the noise of Table 4, as it appears to stem from the same artefact of shrinkage and magnification that jeopardizes amplitude measurements of small saccades. For large saccades, this artefact is likely a major reason why filters need to be employed to smooth velocity profiles such as Fig. 21 before event detection is made (Holmqvist & Andersson, 2017, p. 206).

Our data suggest that when recording data for studying microsaccades, reading and vergence, it would be safest to collect the data with a DPI. Many researchers who have a DPI system have however stopped using it, because of the issues and costs with training and maintenance, and possibly also because of the large post-saccadic oscillations (evident in Fig. 1).

### Outlook

Video-based eye-trackers were originally not made to study small eye-movements. The choice to combine pupil (P) and CR signals to build what has been known as P-CR systems, literally P minus CR, came about to relax requirements on head movement restriction (Holmqvist & Andersson, 2017, p. 77-80).

In recent years, several studies of data quality in eye-trackers have been made by researchers, revealing artefacts in data specifically from video-based eye-tracking. Some of those findings have been frequently replicated, for instance the pupil artefacts (Drewes, 2014; Drewes, Montagnini, & Masson 2011), the post-saccadic oscillations^5^ (Hooge et al., 2016; McCamy et al., 2015), and the effects of movement in the headboxes (Niehorster et al., 2017). Some of these are comparative studies of several eye-trackers (Holmqvist, 2015, e.g.) which not only rate them, but look for common properties.

When the pupil was found to be an unreliable feature in video-based eye-tracking, some authors made the assumption that the CR could be trusted. For instance, Hooge et al. (2016) writes the following about the CR signal being better than the Pupil signal: “spatial resolution of digital high speed cameras still increase ... If one is only interested in saccade dynamics and not so much in absolute gaze direction, CR only eye tracking with the SMI Hi-Speed is a possibility.” The assumption that the CR is a good signal looked reasonable at the time. The CR has a long history in eye-trackers, from Dodge and Cline (1901), via Buswell (1935) and Yarbus (1967), and many more studies from which present eye-movement researchers have learned. However, our data provide evidence that we should not take the CR assumption at face value when the CR is recorded with a video camera.

Pupil-only recordings are available in quite a few high-end eye-trackers and lead to smaller errors than Gaze recordings (Table 8). Pupil-only eye-tracking, for instance with the EyeLink II, would be a possibility for recording less inaccurate small saccades and microsaccades, had it not been for the pupil artefacts. Even when restricting participant heads, amplitude errors will be common for the shortest saccades, whether they are recorded as Gaze, Pupil or CR.

**Fig. 22:**
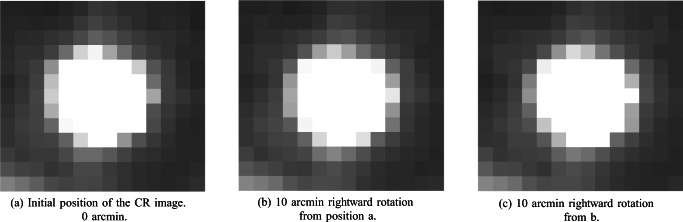
The CR image from three eye photos taken with the eye camera of the SMI HiSpeed 240. In (a) the initial position, then at (b) after a first 10 arcmin rotation, and at (c) another 10 arcmin rotation. The subpixel estimation algorithm determines the center of the CR image in each figure. The distances between the CR centers of (a) and (b) and between (b) and (c) were estimated by the SMI software to be less than 0.06 pixels. If the software makes a miscalculation of the CR centre, an error happens, which translates to an error in the gaze signal.

A major unsolved issue is exactly what it is that causes the errors in the CR signal in modern VOGs. We have shown that it is unlikely that calibration polynomials cause them. Neither does the resolution of the eye camera matter in and by itself. Gaze direction, however, matters. The errors seem to point to mismeasurements of the CR center, in particular the finding in Fig. 9 that 10^′^ rotations correspond to as little as 0.06 pixels movement of the CR in the eye camera. Figure 22 shows how little the camera pixels of the CR differ for 10^′^ rotations. This suggest that sub-pixel estimation is the issue.

Tobii and SR representatives have agreed that sub-pixel estimation of the CR is the likely cause. However, we do not know which sub-pixel estimation algorithms are implemented in commercial VOGs. Center-of-mass algorithms are likely to be common. If manufacturers could rewrite their image processing software to counteract the formation of these errors, then there could be a future for the CR signal of video-based eye-trackers. This, however, assumes that the sub-pixel estimation algorithms can be tweaked without impairment on other quality metrics, such as precision.

Oscillating shrinkage and magnification is common among the VOGs (Table 6), with wavelengths of 30 − 120^′^. But why is there such an oscillation over the measurement space? Is there something in the sub-pixel estimation algorithms that repeats regularly over space?

Another open question is what goes on in data during controlled *smooth* movement. Figure 19 provides an example of a small relative shift between Pupil and CR that coincides with (causes) an artificial retrograde movement in the data that event detectors are likely to flag. There may be more artefacts hiding in VOG data of smooth movements.

Another obvious open question is whether vertical errors are the same as horizontal errors.

We hope to see replications of our study, as well as advances to solve the many questions we have opened, but also the emergence of future eye-trackers with smaller or no amplitude errors, which may or may not be built on video-camera technology.

The Stepperbox exists in two copies: one in Regensburg, Germany and one in Bloemfontein, South Africa. Contact the authors for further details.

## Footnotes

1 Personal communication, Eyal Reingold, Nov 22, 2018.

2 Personal communication, Sam Hutton, Aug-Dec, 2018.

3 Personal communication, Andrew Clarke, Aug. 7, 2012.

4 Because velocity = amplitude / time.

5 Part biology, part artefact.

## References

[CR1] Blignaut, P. (2014). Mapping the pupil-glint vector to gaze coordinates in a simple video-based eye tracker. *Journal of Eye Movement Research, 7*(1), 1–11.

[CR2] Blignaut, P., Holmqvist, K., Nyström, M., & Dewhurst, R. (2014). Improving the accuracy of video-based eye tracking in real time through post-calibration regression. In *Current Trends in Eye Tracking Research* (pp. 77–100) Cham: Springer.

[CR3] Buswell, G. T. (1935). *How people look at pictures*. Chicago: University of Chicago Press.

[CR4] Cerrolaza, J. J., Villanueva, A., & Cabeza, R. (2012). Study of polynomial mapping functions in video-oculography eye trackers. *ACM Transactions on Computer-Human Interaction (TOCHI), 19*(2), 10.

[CR5] Clarke, A. H., Ditterich, J., Drüen, K., Schönfeld, U., & Steineke, C. (2002). Using high frame rate cmos sensors for three-dimensional eye tracking. *Behavior Research Methods, Instruments, & Computers, 34*(4), 549–560. 10.3758/bf03195484 12564559

[CR6] Crane, H., D. & Steele, C. M. (1985). Generation-V dual-Purkinje-image eyetracker. *Applied Optics, 24*(4), 527–537. 10.1364/ao.24.000527 18216982

[CR7] Ditchburn, R., & Ginsborg, B. (1952). Vision with a stabilized retinal image. *Nature, 170*, 36–37. 10.1038/170036a0 14957011

[CR8] Dodge, R.. & Cline, T. S. (1901). The angle velocity of eye movements. *Psychological Review, 8*(2), 145–157.

[CR9] Drewes, J. (2014). Smaller is better: Drift in gaze measurements due to pupil dynamics. *PLoS ONE, 9*(10), e111197. 10.1371/journal.pone.0111197 PMC4206464 25338168

[CR10] Drewes, J., Masson, G. S., & Montagnini, A. (2012). Shifts in reported gaze position due to changes in pupil size: Ground truth and compensation. In *Proceedings of the Symposium on Eye Tracking Research and Applications* (pp. 209–212). New York: ACM.

[CR11] Drewes, J., Montagnini, A., & Masson, G. S. (2011). Effects of pupil size on recorded gaze position: A live comparison of two eye tracking systems. *Journal of Vision, 11*(11), 494–494.

[CR12] Engbert, R.. & Kliegl, R. (2003). Binocular coordination in microsaccades. In *The mind’s eye*, (pp. 103–117).Oxford: Elsevier.

[CR13] Fang, Y., Gill, C., Poletti, M., & Rucci, M. (2018). Monocular microsaccades: Do they really occur? *Journal of Vision, 18*(3), 18–18. 10.1167/18.3.18 PMC5868759 29677334

[CR14] Gautier, J., Bedell, H. E., Siderov, J., & Waugh, S. J. (2016). Monocular microsaccades are visual-task related. *Journal of Vision, 16*(3), 37–37. 10.1167/16.3.37 26913629

[CR15] Holmqvist, K. (2015). Common predictors of accuracy, precision and data loss in 12 eye-trackers. Available at ResearchGate.

[CR16] Holmqvist, K.. & Andersson, R. (2017). *Eye tracking: A comprehensive guide to methods, paradigms and measures*. Lund: Lund Eye-Tracking Research Institute.

[CR17] Holmqvist, K., Nyström, M., & Mulvey, F. (2012). Eye tracker data quality: What it is and how to measure it. In *Proceedings of the Symposium on Eye Tracking Research and Applications* (pp. 45–52). New York: ACM.

[CR18] Hooge, I., Holmqvist, K., & Nyström, M. (2016). The pupil is faster than the corneal reflection (cr): Are video based pupil-cr eye trackers suitable for studying detailed dynamics of eye movements? *Vision Research, 128*, 6–18. 10.1016/j.visres.2016.09.002 27656785

[CR19] Hooge, I. T., Hessels, R. S., & Nyström, M. (2019). Do pupil-based binocular video eye trackers reliably measure vergence? *Vision Research, 156*, 1–9. 10.1016/j.visres.2019.01.004 30641092

[CR20] Hooge, I. T., Niehorster, D. C., Nyström, M., Andersson, R., & Hessels, R. S. (2018). Is human classification by experienced untrained observers a gold standard in fixation detection? *Behavior Research Methods, 50*(5), 1864–1881. 10.3758/s13428-017-0955-x PMC7875941 29052166

[CR21] Ko, H.-k., Snodderly, D. M., & Poletti, M. (2016). Eye movements between saccades: Measuring ocular drift and tremor. *Vision Research, 122*, 93–104. 10.1016/j.visres.2016.03.006 PMC4861686 27068415

[CR22] Kowler, E. (2011). Eye movements: The past 25 years. *Vision Research, 51*, 1457–1483. 10.1016/j.visres.2010.12.014 PMC3094591 21237189

[CR23] Martinez-Conde, S., Macknik, S. L., Troncoso, X. G., & Dyar, T. A. (2006). Microsaccades counteract visual fading during fixation. *Neuron, 49*(2), 297–305. 10.1016/j.neuron.2005.11.033 16423702

[CR24] Martinez-Conde, S., Macknik, S. L., Troncoso, X. G., & Hubel, D. H. (2009). Microsaccades: A neurophysiological analysis. *Trends in Neurosciences, 32*(9), 463–475. 10.1016/j.tins.2009.05.006 19716186

[CR25] McCamy, M. B., Otero-Millan, J., Leigh, R. J., King, S. A., Schneider, R. M., Macknik, S. L., & Martinez-Conde, S. (2015). Simultaneous recordings of human microsaccades and drifts with a contemporary video eye tracker and the search coil technique. *PLOS ONE, 10*(6), 1–20. 10.1371/journal.pone.0128428 PMC4452707 26035820

[CR26] McConkie, G. (1981). Evaluating and reporting data quality in eye movement research. *Behavior Research Methods, 13*(2), 97–106.

[CR27] Niehorster, D. C., Cornelissen, T. H., Holmqvist, K., Hooge, I. T., & Hessels, R. S. (2017). What to expect from your remote eye-tracker when participants are unrestrained. *Behavior Research Methods*, 50, 213–227. 10.3758/s13428-017-0863-0 PMC5809535 28205131

[CR28] Orquin, J. L.. & Holmqvist, K. (2017). Threats to the validity of eye-movement research in psychology. *Behavior Research Methods, 50*(4), 1645–1656. 10.3758/s13428-017-0998-z 29218588

[CR29] Otero-Millan, J., Castro, J. L. A., Macknik, S. L., and Martinez-Conde, S. (2014). Unsupervised clustering method to detect microsaccades. *Journal of Vision, 14*(2), 18. 10.1167/14.2.18 24569984

[CR30] Poletti, M.. & Rucci, M. (2016). A compact field guide to the study of microsaccades: challenges and functions. *Vision Research, 118*, 83–97. 10.1016/j.visres.2015.01.018 PMC4537412 25689315

[CR31] Rayner, K. (1998). Eye movements in reading and information processing: 20 years of research. *Psychological Bulletin, 124*(3), 372. 10.1037/0033-2909.124.3.372 9849112

[CR32] Reingold, E. M. (2014). Eye tracking research and technology: Towards objective measurement of data quality. *Visual Cognition, 22*(3), 635–652. 10.1080/13506285.2013.876481 PMC3996543 24771998

[CR33] Wang, X., Holmqvist, K., ∧ Alexa, M. (2019). The recorded mean point of vergence is biased. *Journal of Eye Movement Research, 12*(4), 2. 10.16910/jemr.12.4.2 PMC7880627 33828744

[CR34] Yarbus, A. L. (1967). *Eye movements and vision*. New York Plenum Press.

[CR35] Zemblys, R., Niehorster, D. C., & Holmqvist, K. (2018a). gazenet: End-to-end eye-movement event detection with deep neural networks. *Behavior Research Methods, 51*(2), 840–864. 10.3758/s13428-018-1133-5 30334148

[CR36] Zemblys, R., Niehorster, D. C., Komogortsev, O., & Holmqvist, K. (2018b). Using machine learning to detect events in eye-tracking data. *Behavior Research Methods, 50*(1), 840–181. 10.3758/s13428-017-0860-3 28233250

